# YOLOv10-based detection of melanocytic nevi: reverse exclusion optimization for melanoma screening

**DOI:** 10.3389/frai.2025.1637842

**Published:** 2025-09-12

**Authors:** ShengJie Wang, Jian Wang, Rui Yin

**Affiliations:** International Sakharov Environmental Institute, Belarusian State University, Minsk, Belarus

**Keywords:** melanoma, melanocytic nevus, deep learning, YOLOv10, reverse exclusion

## Abstract

Malignant melanoma is the deadliest skin cancer, yet its early dermoscopic presentation closely mimics benign melanocytic nevi. Conventional visual or dermoscopic screening therefore suffers from high miss rates and generates excessive biopsies. In this study we focus on Chinese East-Asian patients and introduce a reversed-exclusion strategy—classifying “benign first, exclude malignancy”: lesions that fully meet benign nevus criteria are deemed low-risk; all others are flagged as high-risk. Building on the real-time detector YOLOv10, we incorporate three medical-oriented upgrades: (i) a PP-LCNet backbone to preserve sub-3 mm textures; (ii) a Multiscale Contextual Attention (MCA) neck to enhance cross-scale aggregation; and (iii) a Shape-IoU loss that jointly optimises position, scale, and curvature. The model was trained on a multi-centre dermoscopic dataset from three tertiary hospitals in mainland China (2,040 benign nevi) and independently tested on 365 biopsy-proven melanomas collected at the same medical institution but drawn from a demographically distinct patient cohort, achieving a detection mAP@0.5 of 97.69% for benign lesions and a melanoma false-negative rate (FNR) of only 0.27%. By delivering high-confidence benign identification followed by malignant exclusion, the proposed model offers a high-precision, low-risk pathway for early melanoma screening in Chinese clinical settings. It can markedly reduce unnecessary biopsies while keeping the miss rate below the clinical safety ceiling of 0.5%, thus preserving the life-saving window afforded by early detection.

## 1 Introduction

Malignant melanoma is the deadliest form of skin cancer; when it is diagnosed early, the 5-year survival rate rises from 23% in advanced disease to 99% (Thomas et al., 1998; Whited et al., 1997). Nevertheless, on dermoscopic images early melanomas closely mimic benign melanocytic nevi, so conventional visual inspection or dermoscopy carries a substantial risk of missed diagnosis (Dinnes et al., 2018). Benign nevi are usually symmetrical, uniformly pigmented and sharply demarcated (Lallas et al., 2012); in contrast, even in-situ melanomas reveal subtle asymmetry or focal disruption of the pigment network (Dinnes et al., 2018; Carrera et al., 2017b). Non-melanocytic lesions such as seborrhoeic keratosis and haemangioma display readily distinguishable features, and consequently their clinical confusion rate with melanoma is far lower (Carrera et al., 2017a; Stell et al., 2007). This quasi-mutual exclusivity implies that lesions strictly meeting benign criteria can almost rule out melanoma, whereas any departure from those rules should be regarded as high-risk and sent for biopsy.

The three principal clinical pathways—naked-eye or dermoscopic assessment, periodic imaging follow-up, and surgical biopsy—each have limitations. Large studies show that the ABCDE rule and the seven-point checklist deliver only 65 –80% sensitivity, with inter-observer agreement *κ* < 0.4 (Thomas et al., 1998; Whited et al., 1997), and comprehensive skin screening is constrained by time and workforce. Dermoscopy can raise sensitivity by a further 10 –27% (Dinnes et al., 2018), yet early lesions are often “feature-poor” (Lallas et al., 2012); differences in operator training can produce specificity gaps of ~20 percentage-points (Carrera et al., 2017a), and specificity for non-pigmented lesions remains insufficient, leading to frequent unnecessary biopsies (Carrera et al., 2017b). Biopsy with histopathology—though the diagnostic gold standard—causes scarring and financial burden, and secondary wide excision is required in up to 22% of cases (Stell et al., 2007). Novel non-invasive technologies such as reflectance confocal microscopy achieve ~93% sensitivity and ~76% specificity in large cohorts (Stevenson et al., 2013), but devices cost tens of thousands of US dollars and a single examination may take 10 min (Stevenson et al., 2013); optical coherence tomography demands > 180 scans for novice competence, with systems priced at USD 50000–100,000 (Van Loo et al., 2020; Ferris and Harris, 2012). The quest to combine high sensitivity with an extremely low miss rate therefore remains clinically urgent.

Although deep convolutional networks have advanced medical image analysis, four key bottlenecks impede their direct application to early melanoma detection. First, morphological overlap yields limited separability of high-resolution features (Hunziker et al., 2023). Second, public datasets are dominated by nevus images, inducing class imbalance and model bias (Akash et al., 2023). Third, the pronounced heterogeneity of melanoma morphology necessitates deeper networks and larger receptive fields, raising computational cost and over-fitting risk (Shen et al., 2021; Alzamili and Ruhaiyem, 2025). Fourth, the coarse granularity of pathological labels makes it hard to learn reliable links between malignant behavior and imaging appearance, heightening misclassification of inflammatory or keratotic lesions (Naseri and Safaei, 2025).

**Figure 1:**
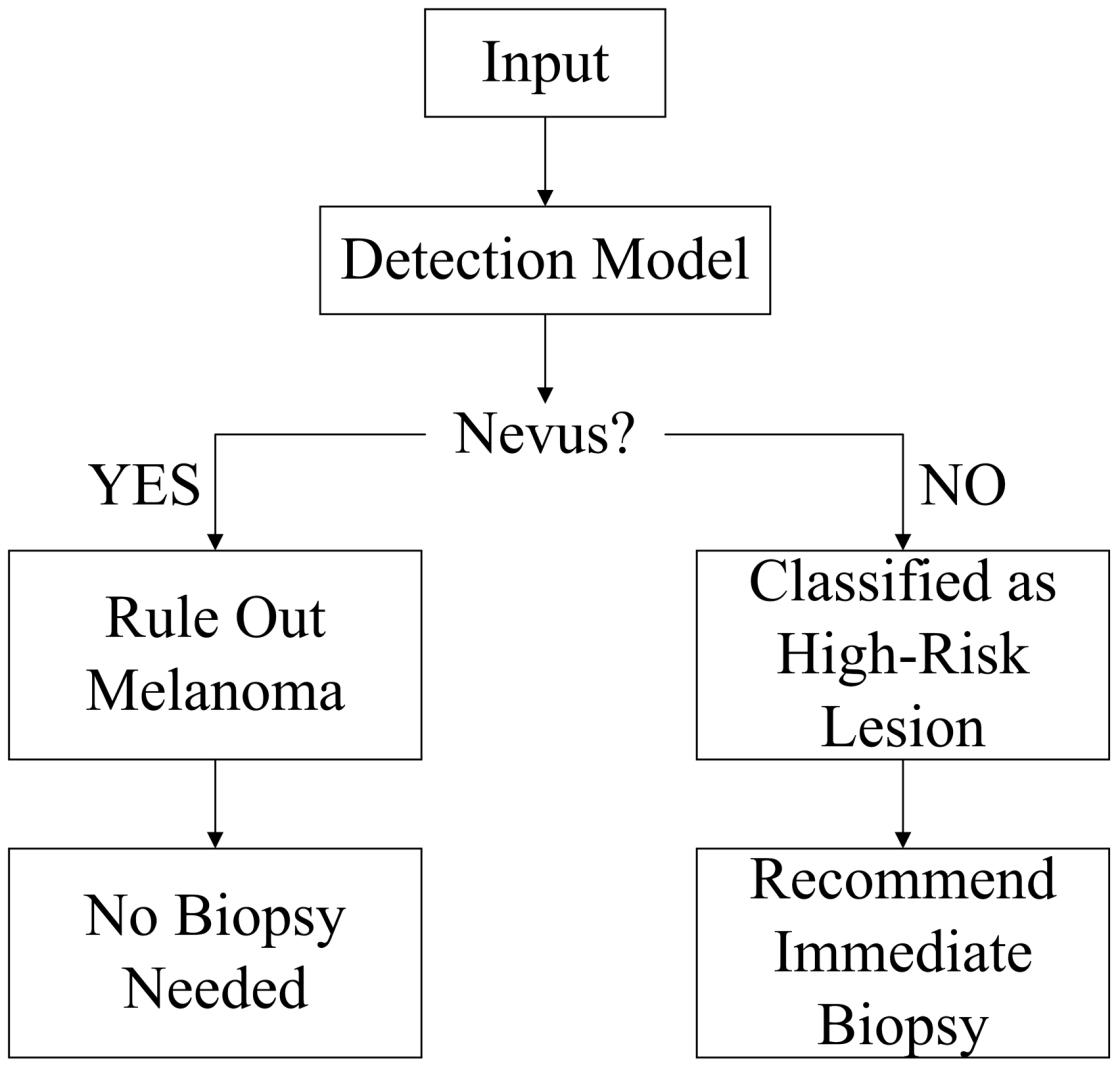
Reverse-exclusion logic diagram for melanoma.

To address these challenges, we propose a “benign-first” inverse-exclusion strategy. Instead of directly trying to detect “malignant” lesions, the model first identifies melanocytic nevi with high confidence if they fully meet benign criteria — symmetry, uniform pigmentation, and sharply defined borders. Any lesion that fails to meet one of these rules is placed in the high-risk category and sent for biopsy. Because early melanoma growth inevitably disrupts at least one of the benign features, this approach naturally suppresses false negatives and enables high-sensitivity screening that adheres to strict clinical safety thresholds. Within the YOLOv10 framework, we incorporate three key enhancements: a lightweight PP-LCNet backbone to preserve sub-3 mm nevus texture, a Multiscale Contextual Attention (MCA) module to capture local irregularities, and a Shape-IoU loss to jointly optimize aspect ratio, centroid position, and border curvature. The model will declare a lesion “melanocytic nevus” only if it fully meets all criteria of symmetry, uniform color, and smooth borders; otherwise, it is flagged as high-risk and referred for biopsy (this workflow is illustrated in Figure 1).

This high-confidence, benign-first workflow strikes a new balance between efficiency and safety, offering a practical technological pathway for early melanoma screening.

## 2 Related work

Early automated melanoma detection relied on handcrafted features such as colour histograms and grey-level co-occurrence matrices; these techniques had limited power for lesions with fuzzy borders, irregular shapes or minute early foci, yielding missed-diagnosis rates of 25 –30% (Almubarak et al., 2017). Even the clinically ubiquitous ABCDE or seven-point scales can show miss rates above 40% when the symmetry index falls below 1.5 (Tsao et al., 2015), with strong operator-dependent variability. The advent of deep learning greatly improved overall accuracy: in the ISIC 2017 challenge, ResNet-based CNNs achieved a mean classification accuracy of 83.7% (Codella et al., 2017), marking a milestone in image pattern recognition. Nonetheless, dataset bias and the scarcity of early lesions persist. In ISIC 2020, histopathology-confirmed melanomas account for only 1.8% of images, leaving models largely untrained on in-situ disease (Rotemberg et al., 2021). A more advanced Vision Transformer reached 92.79% accuracy in multiclass skin-disease classification, yet its melanoma recall remained below 60% (Flosdorf et al., 2024). Clinical studies show that high-quality AI assistance markedly improves diagnostic performance for less-experienced physicians, whereas immature systems may mislead clinicians at all levels (Tschandl et al., 2020), underscoring the need for reliable and interpretable algorithms.

At the detection-framework level, YOLOv10 provides a real-time, high-precision baseline through two-stage dynamic label assignment and a decoupled detection head: official experiments report a 2.8-percentage-point gain in mAP@0.5–0.95 and a further 3.4-point gain for small-object AP (Wang A. et al., 2024); on the Roboflow mole-test the false-negative rate (FNR) fell from 7.6% to 4.1% (Wang A. et al., 2024). Mobile-device tests also showed a 2.3-point rise in binary-classification F1 (Liutkus et al., 2023). Yet the overall FNR still exceeds the clinical safety threshold of 0.5% (Esteva et al., 2017). The root causes are threefold: eight-fold down-sampling leaves nevi < 3 mm represented by only 1–3 pixels, with recall around 68%; a fixed-scale PANet limits small-nevus mAP to roughly 75%; and CIoU is insensitive to curved borders, giving an average 12-point IoU deficit relative to pathology masks, which further dilutes confidence and produces a cascade of low recall and high FNR.

Clinicopathological studies confirm the morphological stability of benign nevi: in biaxial measurements of 56 lesions, 96.4% had bilateral symmetry distances ≤ 0.1 mm (Flotte and Lamberti, 1992); among 209 images, 99.0% showed RGB colour variance < 0.10 (Seidenari et al., 2005); and in 296 cases, 99.3% exhibited edge-gradient values > 0.80 (Fikrle and Pizinger, 2007). Deviations sharply raise malignant risk: in a cohort of 237 followed lesions, those with symmetry or colour heterogeneity had a melanoma positivity rate of 31.6%, odds ratio = 5.7 (95% CI 3.2–9.1) (Arumi-Uria et al., 2003). Conversely, among 430 nevi meeting strict CASH criteria, none transformed over five years, giving an NPV of 100% (95% CI 99.0–100.0%) (Henning et al., 2007). These statistics underpin the strategy of “high-precision benign identification leading to malignant exclusion”.

In summary, neither traditional handcrafted methods nor current deep models can simultaneously deliver the high sensitivity and ultra-low miss rate required for early melanoma detection. YOLOv10’s dynamic matching and decoupled head form a solid base for small-lesion detection, but shallow-layer resolution, cross-scale semantic fusion and boundary regression remain bottlenecks for lowering FNR. Future work must iterate on high-resolution backbones, dynamic multiscale attention and shape-aware loss functions to meet the stringent clinical target of “FNR ≤ 0.5%” in early screening.

## 3 Methodology

### 3.1 Melanocytic nevus detection model based on improved YOLOv10

#### 3.1.1 Analysis of YOLOv10 baseline structure and limitations in medical detection accuracy

**Figure 2:**
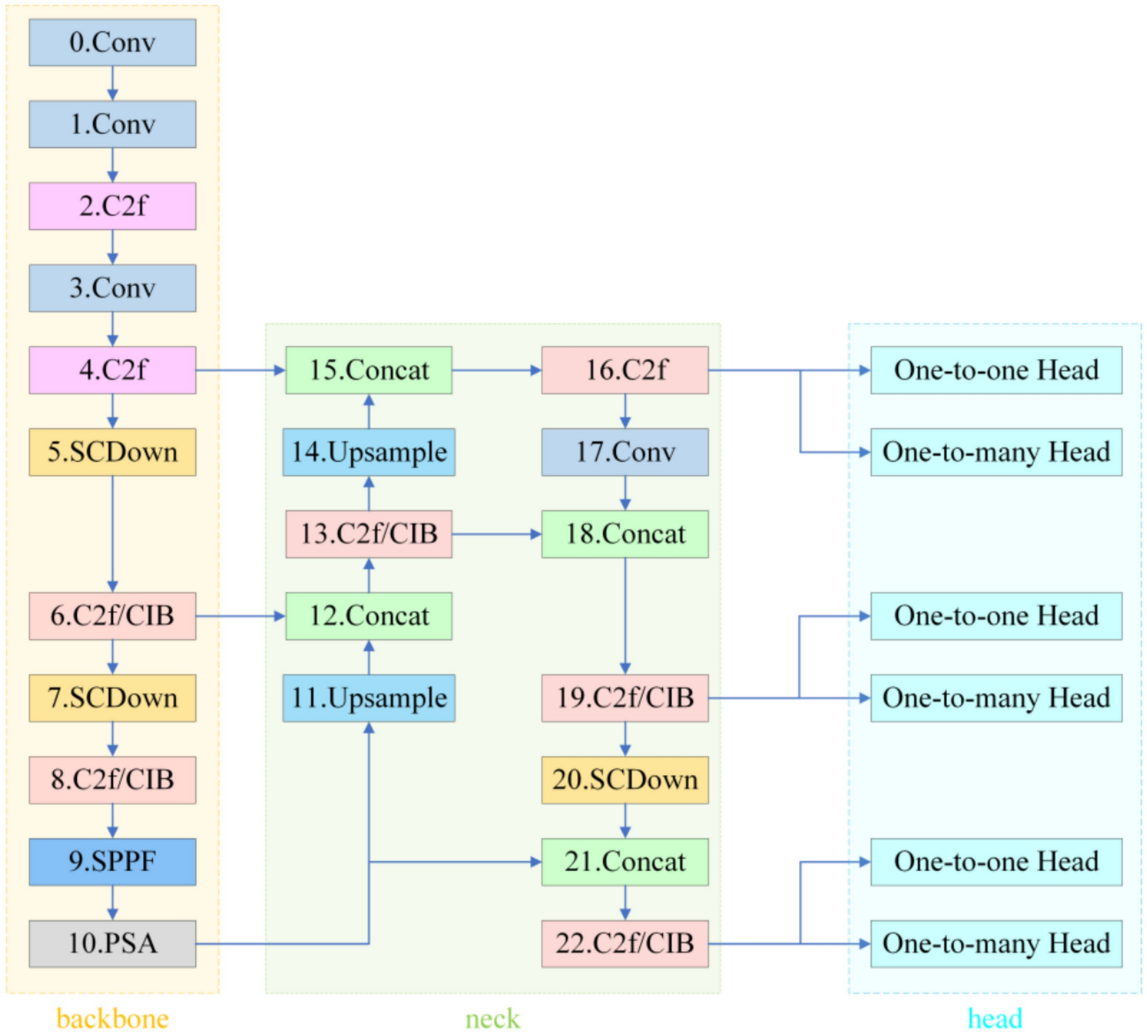
YOLOv10.

As an end-to-end detection framework, YOLOv10 adopts the classical three-stage Backbone–Neck–Head architecture (see Figure 2).

The backbone, CSPDarknet-Tiny, completes 8 × spatial down-sampling in its first two stages and enlarges the receptive field with SPPF and PSA modules; however, this early compression leaves micro-nevi (< 3 mm) occupying only 1–3 pixels on the feature maps, erasing fine pigment patterns. In a study of 59,090 dermoscopic images, Liutkus et al. reported an average recall of just 68.0% for such lesions (Wang A. et al., 2024).

In the Neck, the fixed three-level pyramid inherited from PANet merges scales through repeated up-sampling and concatenation but lacks an adaptive receptive-field mechanism for multi-scale coexisting lesions, limiting the mAP@0.5 for small nevi to 75.0% (Wang A. et al., 2024) and thus constraining high-confidence “benign-first” screening. The decoupled head combines SimOTA and Task-Aligned Assigner to ease gradient coupling, yet the regression branch relies on the default CIoU loss; CIoU is insensitive to curved edges and degenerates to IoU as a prediction box nears its ground truth (Cai et al., 2023). Rectangular boxes therefore fit circular or mildly curved lesions much worse than shape-sensitive losses (Nguyen et al., 2022). Empirically, the average IoU (or mAP) gap between predictions and pathological annotations reaches 10–12 percentage points (Ye et al., 2024), further depressing classification confidence and heightening miss-detection risk (Annadatha et al., 2022).

In summary, YOLOv10 exhibits three critical bottlenecks in melanocytic nevus detection: insufficient shallow-layer resolution, rigid cross-scale modeling, and morphologically inaccurate boundary regression.

#### 3.1.2 Medical adaptation of lightweight backbone network PP-LCNet

**Figure 3:**
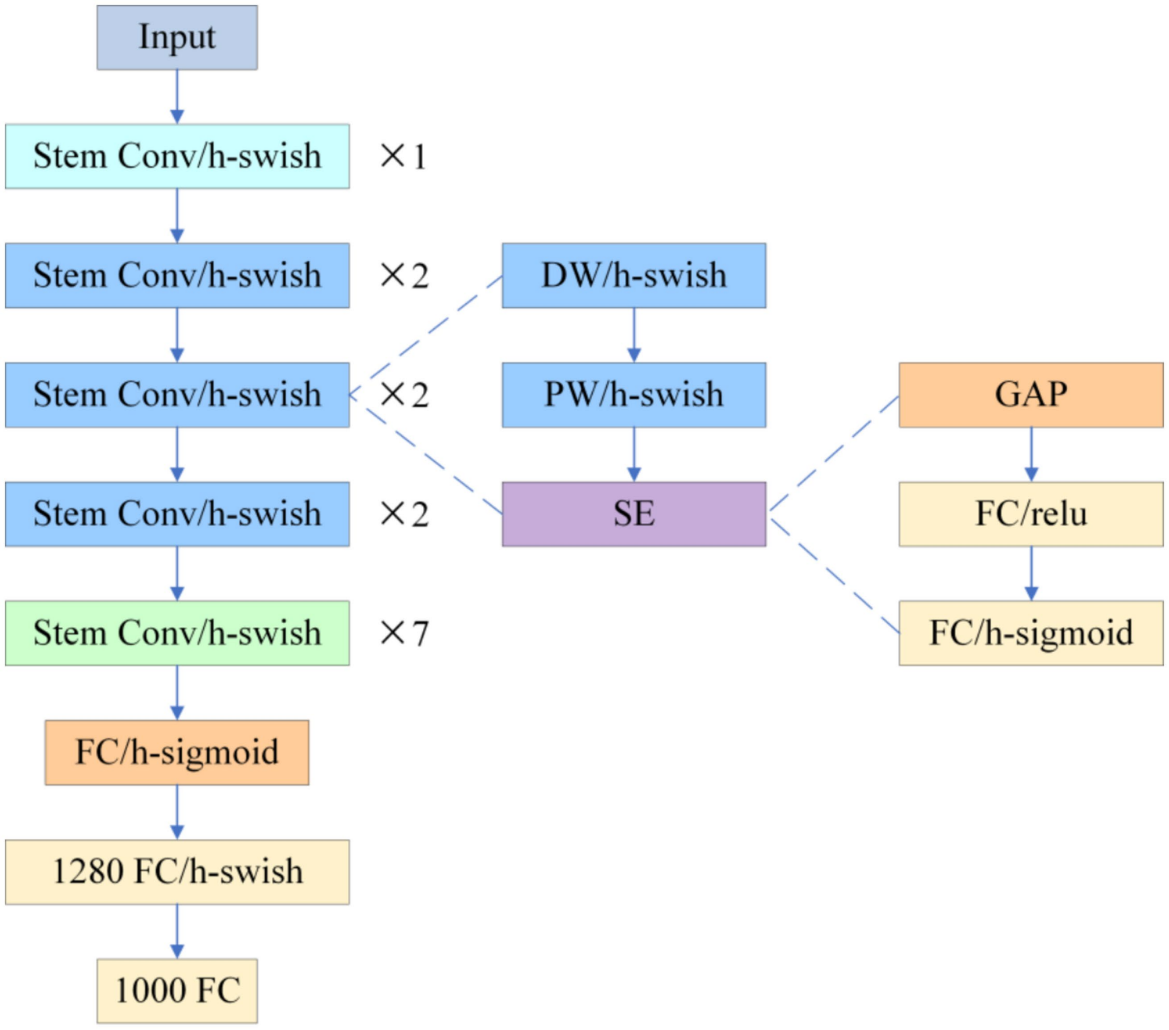
PP-LCNet.

To compensate for CSPDarknet-Tiny’s excessive compression of shallow textures in micro-nevi (< 3 mm), this study replaces the YOLOv10 backbone with PP-LCNet (see Figure 3).

The network begins with a Stem Conv and multi-stage depthwise-separable convolutions, appends a stack of 5 × 5 large-kernel convolutions at the tail, employs H-Swish activation and Squeeze-and-Excitation (SE) throughout, and inserts a 1 × 1 convolution with 1,280 channels after global average pooling to reinforce global aggregation. Experiments show that switching ReLU to H-Swish alone increases ImageNet-1 k Top-1 accuracy from 55.58 to 58.18%; adding SE lifts it to 59.91%, and the combination of 5 × 5 kernels with the high-dimensional 1 × 1 convolution pushes accuracy to 63.14%, clearly outperforming MobileNetV2 (53.21%) and ShuffleNetV2 (53.73%) at comparable scales (Cui et al., 2021). When transferred to dermoscopic analysis, PP-LCNet’s heightened sensitivity to faint pigment networks and blurred boundaries markedly lowers micro-nevus miss rates and improves boundary discrimination, delivering higher-confidence support for the subsequent “benign-first, malignancy-exclusion” workflow without a significant increase in computational cost.

#### 3.1.3 Medical adaptation of lightweight backbone network PP-LCNet

**Figure 4:**
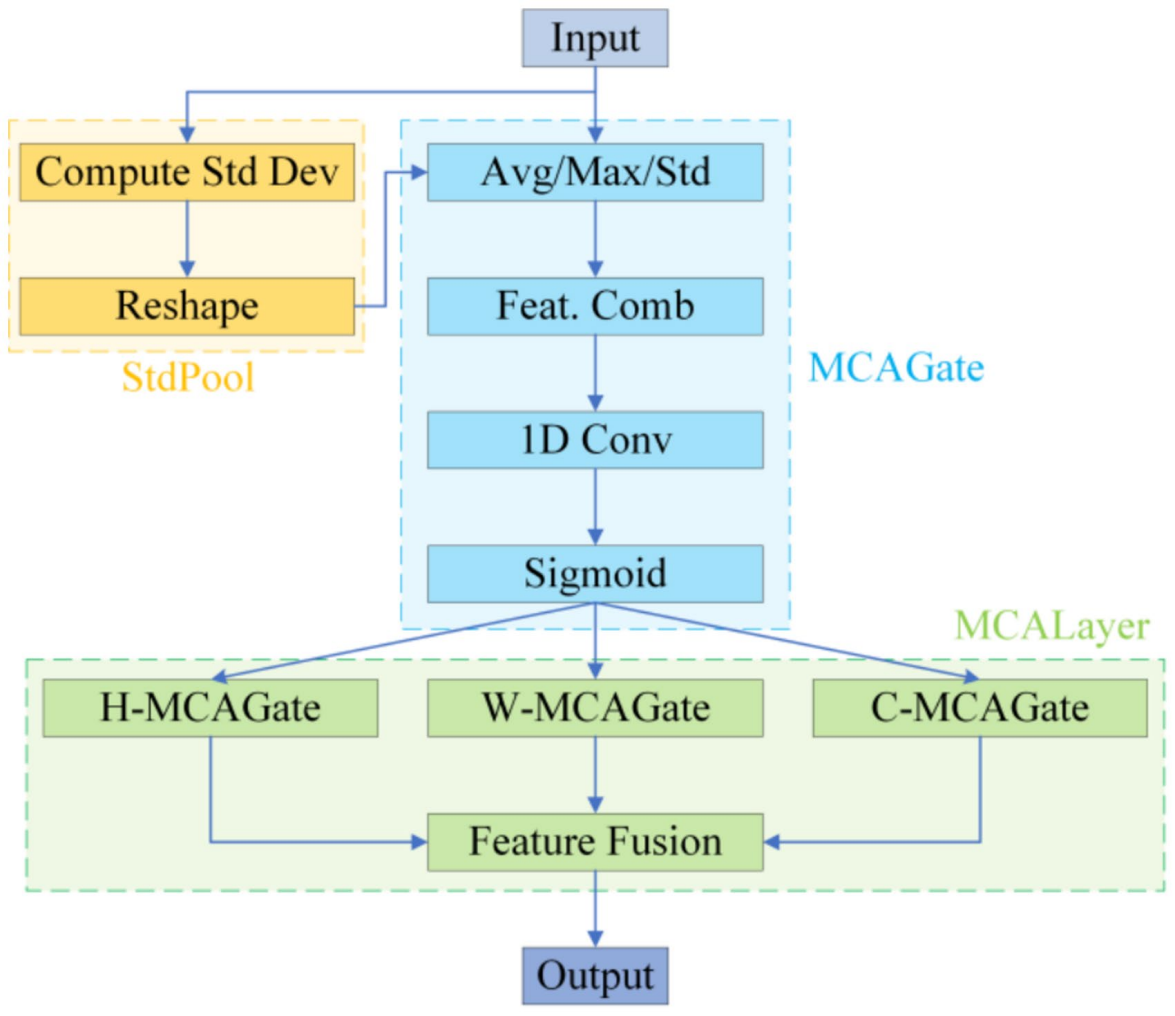
MCA structure diagram.

To remedy the limited contextual parsing of micro-nevi caused by PANet’s fixed-scale fusion, we replace the entire attention branch in YOLOv10’s neck with a Multidimensional Collaborative Attention (MCA) module (see Figure 4).

**Figure 5:**
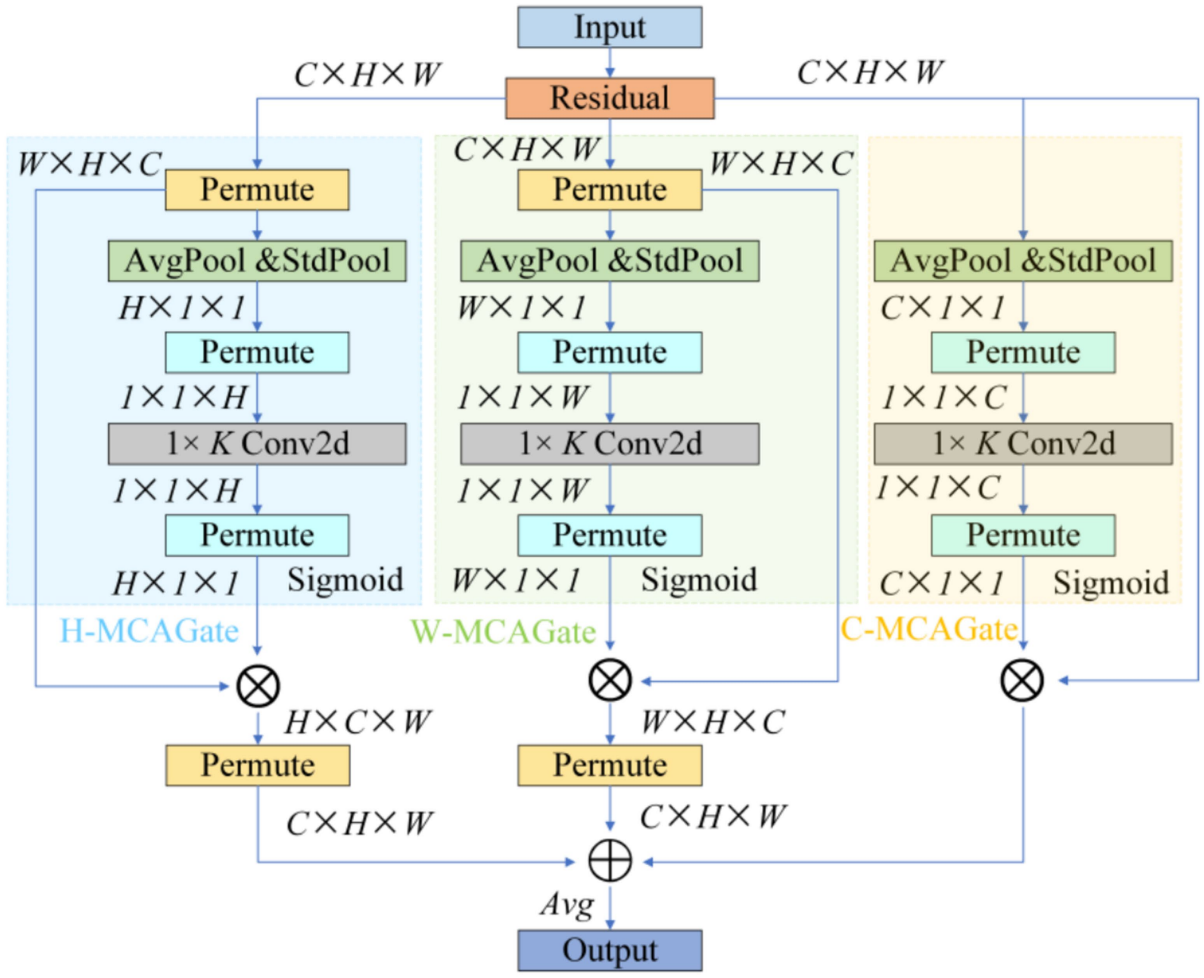
MCA layer.

MCA begins by applying average, standard-deviation and max pooling to the input features in parallel, using the three statistics to construct a complementary brightness–texture–extremum background. During the “squeeze” phase, learnable weights dynamically balance the contributions of average and standard-deviation pooling, while the “excitation” phase substitutes the SE-MLP reduction with 1 × k grouped convolutions to prevent channel-information mismatch; this strategy yields a 1.8 –2.6% Top-1 accuracy gain on ImageNet-1 k (Yu et al., 2023). The module then models attention independently along channel, width and height dimensions, averages the Sigmoid-normalised weights to suppress single-branch noise amplification, and thus simultaneously answers “where to look” and “what to look for” (see Figure 5).

This “multi-pooling context – adaptive fusion – three-dimensional collaboration” scheme markedly increases sensitivity to < 3 mm, low-contrast lesions, lowers the miss rate for small nevi, and delivers the precision needed to support the subsequent “benign-first, malignancy-exclusion” screening framework (Yu et al., 2023).

#### 3.1.4 Medical adaptability improvement of shape-sensitive loss function shape-IoU

The default CIoU regression in YOLOv10 is insensitive to curved or mildly convex edges, yielding an average 12-percentage-point IoU gap between predicted boxes and pathological ground truth and thus degrading classification confidence while elevating miss risk (Zhang and Zhang, 2023). We therefore introduce Shape-IoU, which replaces a purely overlap-based metric with a three-factor model—similarity, shape deviation, and scale sensitivity. While retaining IoU as the structural-consistency baseline, Shape-IoU adds horizontal and vertical weights *w_w_* and *h_h_* to capture width-to-height proportionality, and normalises the centre–distance penalty by the convex-hull diagonal so that “centre offset” is scaled to lesion size. An additional shape-difference term *Ω^shape^* applies an exponentially decaying weight to width–height discrepancies, enforcing tighter fits for targets with regular boundaries and stable aspect ratios (e.g., benign nevi). This loss suppresses “large-box dominance,” lowers the chance that slightly misaligned early melanomas are labelled benign, and markedly improves localisation accuracy to meet the clinical low-miss-rate requirement.

**Figure 6:**
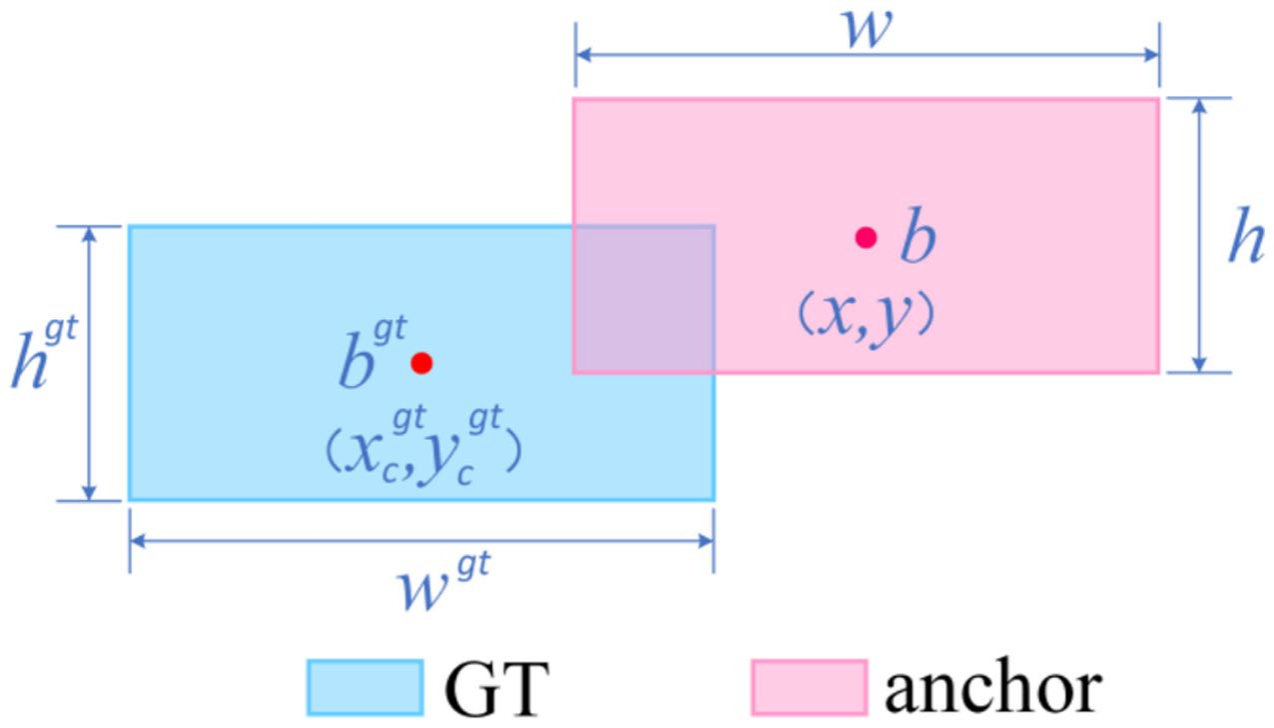
Shape-IoU loss function formula inference diagram.

The specific formula (Equations 1–7) be derived from Figure 6.

(1) $$\mathrm{IoU}=\frac{|B\cap B^{\mathrm{gt}}|}{|B\cup B^{\mathrm{gt}}|}$$

(2) $$w_{w}=\frac{2\times {\left(w^{\mathrm{gt}}\right)}^{\text{scale}}}{{\left(w^{\mathrm{gt}}\right)}^{\text{scale}}+{\left(h^{\mathrm{gt}}\right)}^{\text{scale}}}$$

(3) $$h_{h}=\frac{2\times {\left(h^{\mathrm{gt}}\right)}^{\text{scale}}}{{\left(w^{\mathrm{gt}}\right)}^{\text{scale}}+{\left(h^{\mathrm{gt}}\right)}^{\text{scale}}}$$

(4) $$\text{distanc}e^{\text{shape}}=\mathrm{hh}\times {\left(x_{c}-x_{c}^{\mathrm{gt}}\right)}^{2}/c^{2}+\mathrm{ww}\times {\left(y_{c}-y_{c}^{\mathrm{gt}}\right)}^{2}/c^{2}$$

(5) $$\Omega^{\text{shape}}=\sum_{t=w,h}\;{\left(1-e^{-\omega_{t}}\right)}^{\theta },\theta =4$$

(6) $$\left\{ \begin{array}{c} \omega_{w}=\mathrm{hh}\times \frac{|w-w^{\mathrm{gt}}|}{\max(w,w^{\mathrm{gt}})}\\ \omega_{h}=\mathrm{ww}\times \frac{|h-h^{\mathrm{gt}}|}{\max(h,h^{\mathrm{gt}})}\;\; \end{array}\right.$$

Where, scale is the scale factor reflecting the target’s actual size in the dataset; $\omega_{x}$ and $h_{x}$ are the horizontal and vertical weighting coefficients, respectively, whose values are closely related to the width-height shape of the ground truth (GT) box. Based on this definition, the corresponding bounding box regression loss function can be formulated as:

(7) $$L_{\text{Shape}-\mathrm{IoU}}=1-\mathrm{IoU}+\text{distanc}e^{\text{shape}}+0.5\times \Omega^{\text{shape}}$$

**Figure 7:**
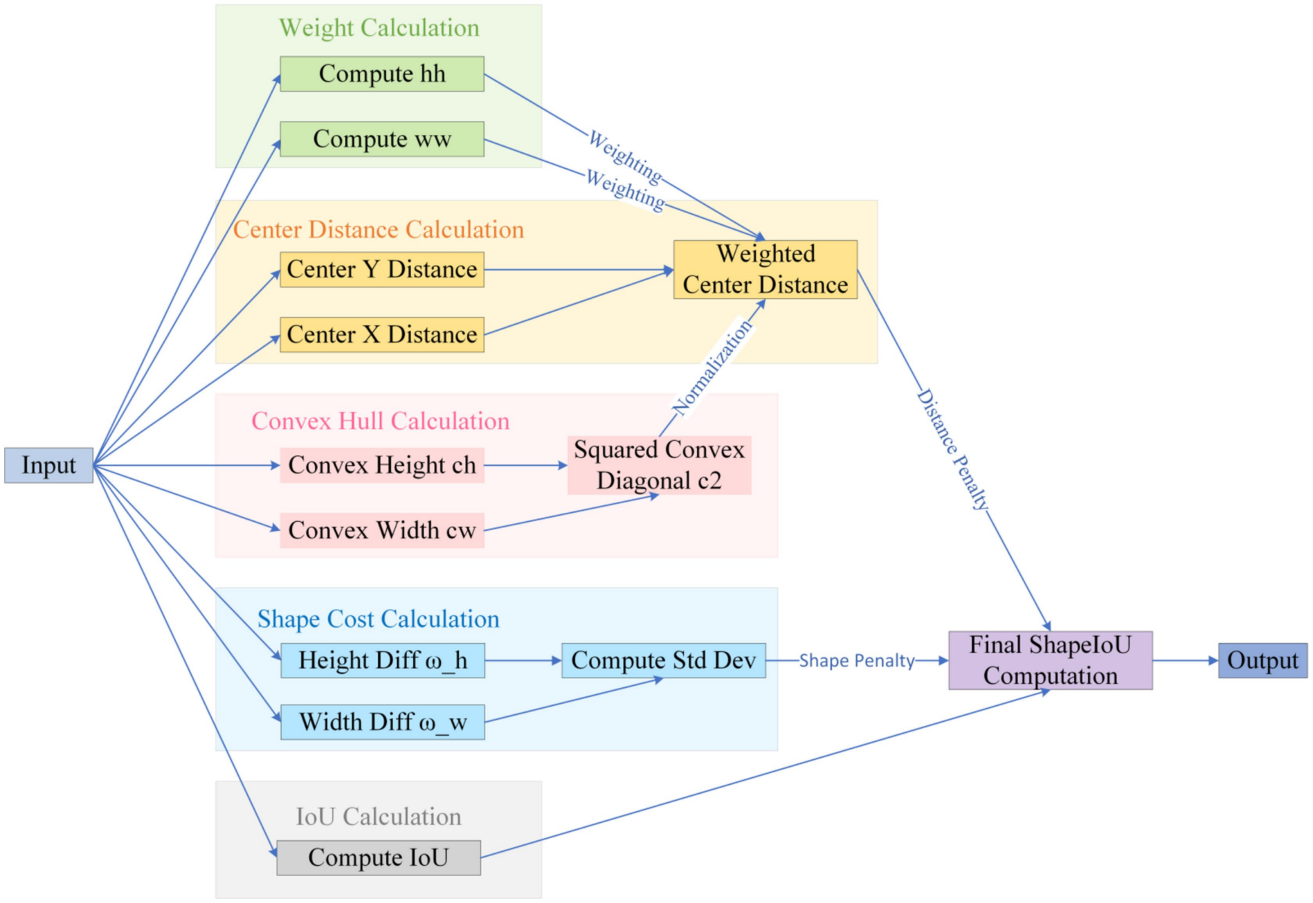
Shape-IoU logic diagram.

The specific logic is illustrated in Figure 7.

In summary, Shape-IoU simultaneously addresses positional, scale, and shape discrepancies, overcoming the insensitivity of generic IoU-series losses to arc-shaped boundaries in medical imaging. This approach is expected to enhance the synergistic accuracy of localization and classification.

#### 3.1.5 Overall model architecture

**Figure 8:**
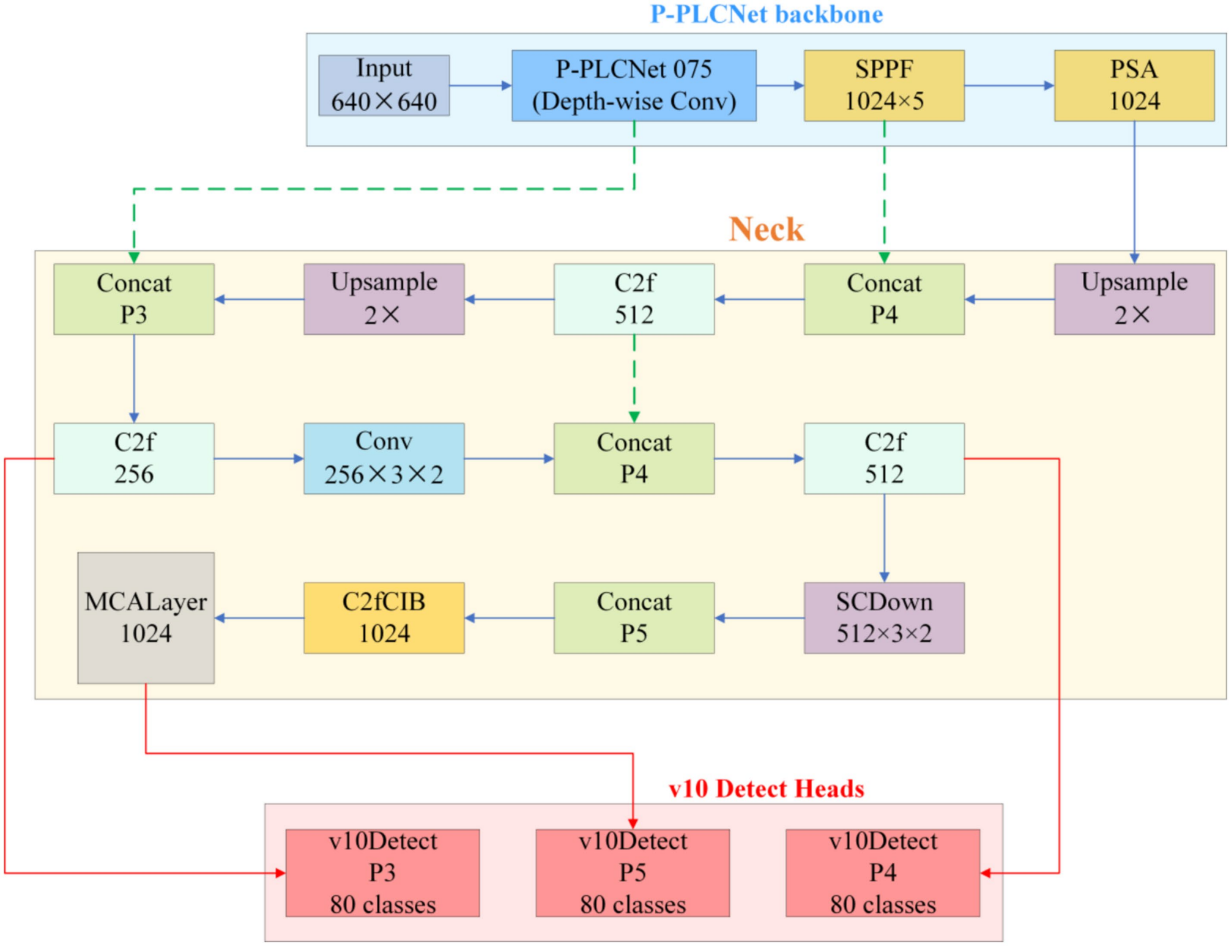
YOLOv10 + PP-LCNet+MCA + shapIoU overall structure.

The improved detection network retains YOLOv10’s single-stage end-to-end framework but sequentially integrates precision-enhancing modules tailored for melanocytic nevus characteristics across three critical layers—backbone, neck, and detection head—forming a high-resolution information chain from shallow to deep levels. The specific network structure is shown in Figure 8.

The internal information flow is divided into three segments—texture fidelity, multiscale aggregation, and shape-sensitive regression—progressively resolving the original model’s detection bottlenecks for small nevus targets.

##### 3.1.5.1 Stage 1: texture fidelity

A 640 × 640 px dermoscopic image enters the PP-LCNet-based backbone. Starting with a 3 × 3/2 Stem Conv to open a high-resolution channel, the backbone sequentially stacks seven groups of depthwise separable convolutions. The first four groups remain at 1/4 and 1/8 resolutions, preserving pixel-level textures of nevi smaller than 3 mm in diameter. After outputting 1/16 and 1/32 feature layers, the backbone expands the receptive field via 5 × 5 SPPF and recalibrates global channels using SE-PSA. This yields three complementary multiscale feature maps: P3 (1/8), P4 (1/16), and P5 (1/32).

##### 3.1.5.2 Stage 2: multiscale aggregation

The three feature maps enter the modified PANet. The network first upsamples P5 by 2 × and concatenates it with P4, generating P4′ after C2f fusion. P4′ is then upsampled and concatenated with P3 to produce P3′. Concurrently, P3′ and P4′ are downsampled via 3 × 3/2 SCDown to deeper layers, enhancing semantic density to form P4″ and P5″. Before these nodes (P3′, P4″, P5″), the system inserts Multiscale Contextual Attention (MCA) layers. MCA first performs parallel average pooling and standard deviation pooling on input features to encode luminance and texture dispersion. It then independently computes attention weights across channel, width, and height branches, dynamically mapping them to the 0–1 range via Sigmoid. The three-dimensionally weighted features—denoted as M-P3, M-P4, M-P5—carry context representations optimized for small, low-contrast lesions.

##### 3.1.5.3 Stage 3: shape-sensitive regression

The calibrated features are fed into the decoupled v10Detect Head. While retaining YOLOv10’s three-branch structure, the detection head replaces CIoU with Shape-IoU for boundary regression. The new loss introduces scale weights, center distance penalties, and aspect ratio difference terms alongside IoU, providing steeper gradient feedback for arc-shaped or mildly curved boundaries. During training, “one-to-one” and “one-to-many” dual-branch label assignments operate in parallel. During inference, only the one-to-one branch is retained, directly outputting final detection boxes and class confidences from M-P3, M-P4, and M-P5 scales without relying on NMS post-processing.

### 3.2 Multicenter dataset construction

#### 3.2.1 Data sources and image annotation standards

The primary training data for this study were collected from three tertiary Grade-A hospitals under the same medical institution, ensuring horizontal consistency in imaging protocols and equipment parameters. The geographic and temporal distribution of the three hospital branches is as follows Table 1.

**Table 1:** Data sources.

Campus	Collection period	Sample size	Imaging equipment	Remarks
Anhui Medical University First Affiliated Hospital—Changjiang Road Campus	2018–2023	702	DermLite DL4 + HD camera	Routine dermatology follow-up
Anhui Medical University First Affiliated Hospital—High-Tech Zone Campus	2020–2024	1,062	Same as above	New campus, same model equipment
Anhui Medical University First Affiliated Hospital—Northern Campus	2022–2024	276	Same as above	Standardized imaging protocol

A total of 2,040 dermoscopic images were confirmed as benign melanocytic nevi through clinical examination.

**Figure 9:**
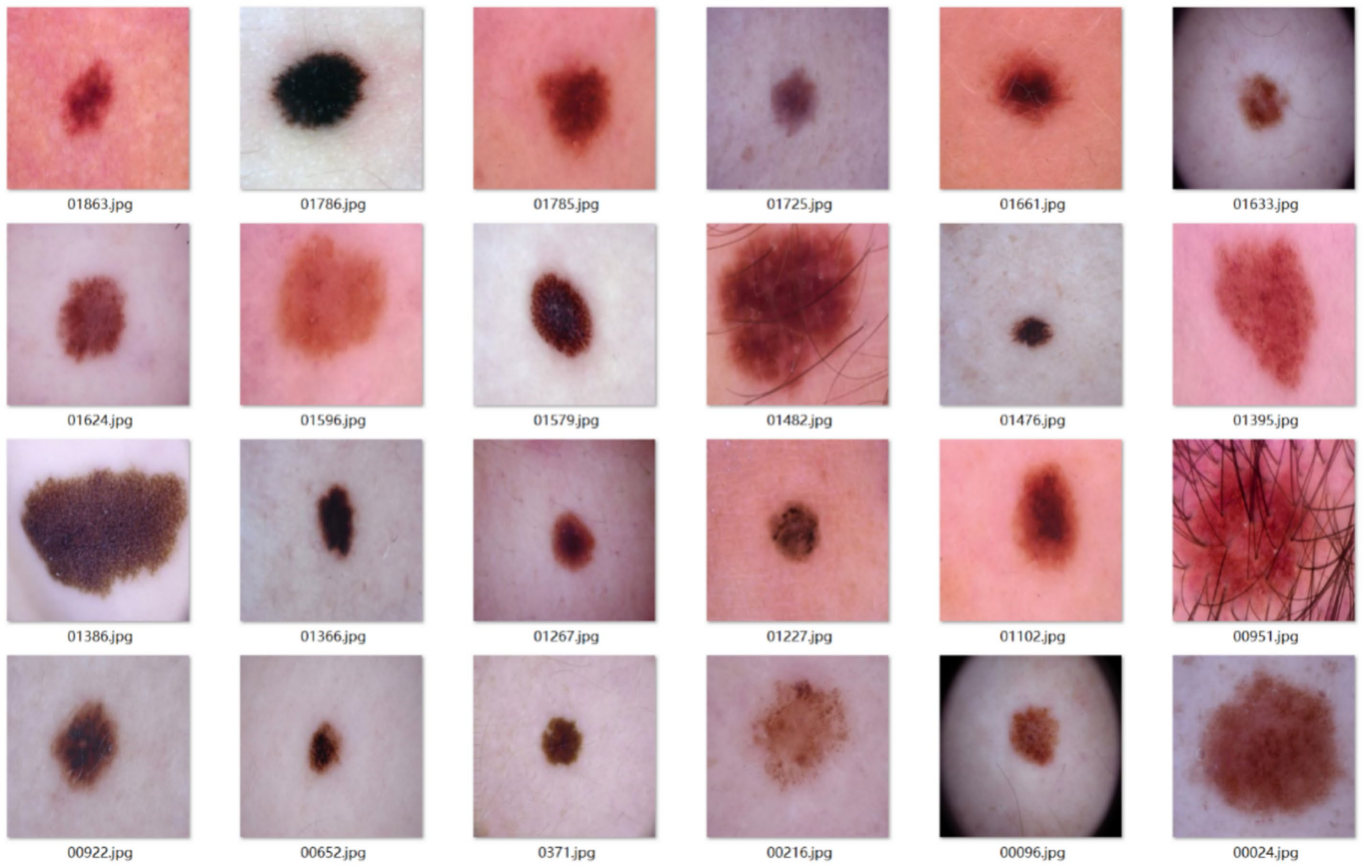
Example images of the melanocytic nevi dataset.

Sample images from the main dataset are shown in Figure 9.

Given that nevus lesions are not routinely biopsied in standard clinical care, the gold standard for this dataset was established through a clinical double-blind assessment with expert adjudication protocol. All patient-identifiable data were rigorously scrubbed from the collected records, including:

1. Initial screening and acquisition: patients underwent standardized imaging using 10–20 × polarized dermoscopy during outpatient visits or follow-ups, adhering to institutional protocols. Images with glare or focal plane deviations were immediately reacquired.
2. Double-blind evaluation: two dermatologists with attending physician or higher qualifications independently interpreted anonymized images. They applied ABCD scoring and benign nevus morphological criteria (symmetry, color homogeneity, sharp borders) to classify lesions as “nevus” or “non-melanocytic nevus”.
3. Expert arbitration: discrepant evaluations were reviewed by a third senior specialist (associate chief physician or above). Images unresolved by arbitration were excluded.
4. Quality control: retained images met strict criteria: clear focus, uniform illumination, and no significant artifacts.

Through this workflow, the study constructed a main dataset of 2,040 fully benign melanocytic nevi, providing reliable imaging and labeling foundations for training the “high-confidence nevus identification → inverse melanoma exclusion” model.

#### 3.2.2 External test set construction

To independently validate the inverse exclusion model’s reliability on malignant lesions, this study separately constructed an external test set containing exclusively early-stage melanomas.

**Figure 10:**
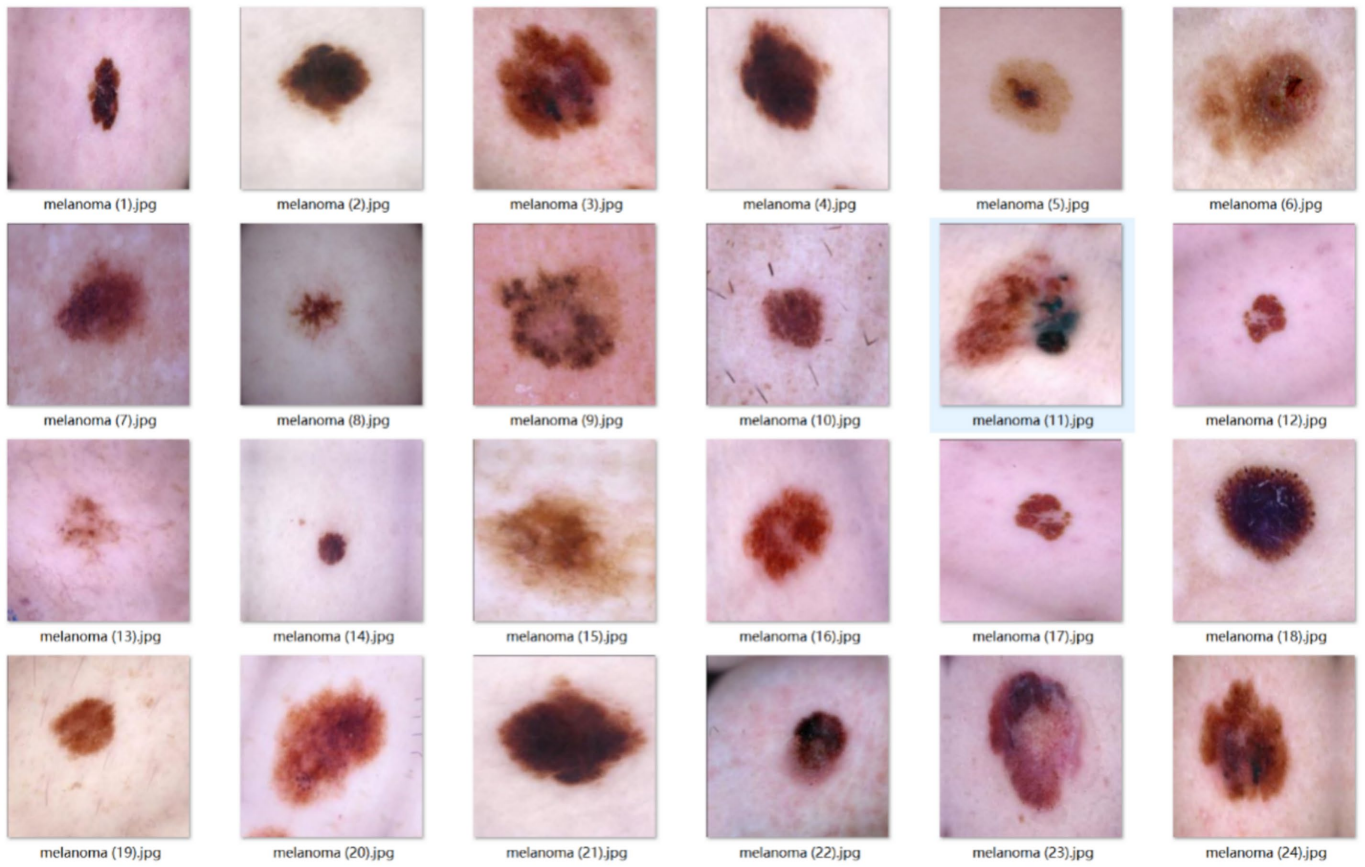
Early-stage melanoma dataset example images.

Sample images from the external test set are shown in Figure 10.

The dataset was collected and selected according to the following rationale:

First, all images originate from the same sources as the main benign-nevus dataset, ensuring identical imaging equipment, acquisition protocols, and time span. This eliminates cross-device bias and guarantees that malignant and benign images share the same temporal context.

Second, every case satisfies the pathological gold standard. Only images from lesions surgically excised or biopsied and definitively diagnosed as “melanoma” in the pathology report are included. Any image lacking biopsy confirmation or based solely on clinical judgment is excluded to avoid label-related evaluation bias.

Finally, all patient identifiers were removed. Every record was fully anonymized and renumbered in random order, ensuring strict data privacy.

After this screening, 365 dermoscopic images of biopsy-positive melanomas were compiled. They serve exclusively as an independent test set after model training and are not used in any training or hyper-parameter tuning. This design directly evaluates whether, within the inverse-exclusion workflow, the model can correctly classify every malignant lesion as “non-melanocytic nevus,” thereby verifying that the false-negative rate (FNR) meets the clinically acceptable threshold.

#### 3.2.3 Participant skin phototype and hair-occlusion characteristics

All 2,405 dermoscopic images (2,040 benign nevi; 365 melanomas) were obtained from a Chinese East-Asian cohort. Two board-certified dermatologists independently assessed Fitzpatrick skin type, hair-occlusion grade, and anatomical site under double-blind conditions; disagreements were adjudicated by a third expert (Cohen’s *κ* = 0.92). Hair interference was first screened with the DullRazor algorithm and then manually reviewed. Only images meeting UQI > 0.95 and NIQE < 5.0 were retained. Table 2 summarises the dataset.

**Table 2:** Population characteristics of the dermoscopic dataset.

Variable	Levels	n	Total
Fitzpatrick skin type	II	1,115	46.36%
	III	1,290	53.64%
	I/IV/V/VI	0	0%
Hair-occlusion grade	0 — none	2,258	93.89%
	1 — sparse vellus hair	104	4.32%
	2 — dense terminal hair	43	1.79%
Anatomical site	Extremities	1,176	48.90%
	Trunk	929	38.60%
	Head/face/neck	300	12.50%

Skin phototypes are concentrated in Fitzpatrick types II–III. Total hair occlusion accounts for only 6.11%, with dense terminal hair representing less than 1.79%, suggesting negligible interference with image quality or feature extraction. Although 17 anatomical sites are represented, the study targets the dermoscopic phenotype of early melanoma; site information was recorded solely to ensure balanced sampling. Owing to the ethnic homogeneity, narrow skin-type range, minimal hair interference, and uniform site distribution, additional stratification of the dataset was not required. However, this uniformity implies that the dataset—and any models trained on it—are presently applicable only to East-Asian populations with Fitzpatrick II–III skin types; external validation across diverse skin tones and ethnic backgrounds will be essential before broader clinical deployment.

### 3.3 Experimental design and environment configuration

This chapter assesses the enhanced model (YOLOv10 + PP-LCNet + MCA + Shape-IoU) with ablation and comparative experiments: individual modules are removed sequentially to gauge their standalone impact, after which the complete model is benchmarked against the baseline to confirm aggregate gains. Each experiment alters only one module and is executed under identical hardware, software, and hyper-parameter settings to guarantee trustworthy conclusions.

#### 3.3.1 Definition of evaluation metrics

This section evaluates model performance with a unified threshold and multiple metrics. A detection is labeled positive when its confidence is ≥ 0.5; it counts as a true positive (TP) only if the predicted box has IoU ≥ 0.5 with the ground-truth box and the class is correct, otherwise it is a mis-detection. The primary metric is mAP@0.5, supplemented by mAP@0.95, precision, recall, model size (Wsz, MB), and inference Latency (ms). Here, mAP@0.5 is the mean of class-wise average precision (AP) at IoU = 0.5, while mAP@0.95 is the mean AP computed from IoU 0.50 to 0.95 in 0.05 steps, reflecting bounding-box localization accuracy (Song and Wang, 2025). The 0.5 confidence threshold balances higher precision with adequate recall, providing a comprehensive measure of melanocytic-nevus detection performance.

#### 3.3.2 Experimental environment configuration and training process

A single high-performance GPU server was used for hardware infrastructure, with computational and storage resources meeting the requirements for 640 × 640 pixel input at batch size 64 (Table 3).

**Table 3:** Experimental hardware configuration.

Component	Specification	Description
GPU	NVIDIA A100 40 GB (PCIe)	Single-card training and inference
CPU	Intel Xeon silver 4210R (10-core 2.4 GHz)	Data loading and post-processing
Memory	256 GB DDR4	Avoids memory bottlenecks during bulk loading
Storage	1 TB NVMe SSD	Enhances data I/O throughput

The software stack was standardized on Ubuntu 20.04 + Python 3.9 + CUDA 10.1 + PyTorch 1.10.1. Core dependencies are listed in Table 4.

**Table 4:** Core software and library versions.

Library/tool	Version	Function
Ubuntu	20.04LTS	64-bit OS
Python	3.9	Runtime environment
PyTorch	1.10.1	Deep learning framework
CUDA	10.1	GPU computing platform
Torchvision	0.11.1	Image processing & model components
OpenCV	≥4.6.0	Low-level image preprocessing
Pandas	≥1.1.4	Result statistics & analysis
Seaborn	≥0.11.0	Result visualization

All ablation and comparative models adhered to the unified hyperparameter baseline in Table 5, ensuring performance variations originated solely from network architecture differences.

**Table 5:** Training parameter baseline.

Parameter	Setting
Input size	640 × 640 px (RGB)
Batch size	64
Epochs	100
Optimizer	SGD (Momentum 0.9, Weight Decay 5e-4)
Learning rate schedule	Cosine Decay: 0.01 → 1e-5
Early stopping	Stop if validation mAP@0.5 shows no improvement for 15 epochs

The complete training and evaluation process is as follows:

(1) Data preparation

The main dataset (2,040 nevus images) was split into training (1,836) and validation (204) sets at a 9:1 ratio by patient ID.

Pixel values were normalized to [0, 1], followed by channel-wise standardization using ImageNet mean (0.485, 0.456, 0.406) and standard deviation (0.229, 0.224, 0.225) (Krizhevsky et al., 2017).

(2) Online data augmentation

Training-phase augmentations included geometric, photometric, noise, and occlusion categories, as shown in Table 6.

(3) Training process

**Table 6:** Data augmentation method.

Category	Operation	Parameter Range	Purpose
Geometric	Horizontal flip	*p* = 0.5	Eliminate left–right orientation bias
	Vertical flip	*p* = 0.3	Adapt to varying capture angles
	Random scaling	0.5–1.2×	Expand size distribution
Photometric	HSV jitter	H ± 20%, S ± 30%, V ± 30%	Resist lighting/color temperature variations
Noise	Gaussian noise	σ = 0.1	Simulate sensor noise
Occlusion	Random rectangular	Area ≤20%, AR 0.5–2	Replicate hair and glare artifacts

Models were trained for 100 epochs using hyperparameters from Table 5.

Validation set mAP@0.5 was evaluated every 5 epochs; training halted early if no improvement occurred over 15 epochs.

(5) Inference and evaluation

Images underwent standardization and aspect-ratio-preserving padding without random perturbations.

A unified confidence threshold of 0.5 was applied; YOLO series disabled NMS to maintain end-to-end properties, while SSD retained default NMS.

The checkpoint with highest validation mAP@0.5 was selected as the final model for Chapter 6 results reporting.

This workflow ensured full alignment across hardware, software, data augmentation, and hyperparameters, establishing a strict and reproducible experimental baseline for subsequent ablation and comparative studies.

#### 3.3.3 Ablation experiment design

To isolate the independent contributions of the PP-LCNet backbone, MCA attention mechanism, and Shape-IoU loss function to model performance, five ablation experiments were designed by incrementally integrating improvement modules.

Table 7 presents the model combination schemes for the ablation study, where only a single module is introduced at a time to isolate its impact.

**Table 7:** Ablation experiment module configuration.

Model variant	PP-LCNet	MCA	Shape-IoU	Description
YOLOv10 (baseline)	–	–	–	Original YOLOv10 architecture
YOLOv10 + PP-LCNet	✓	–	–	Backbone network replacement only
YOLOv10 + MCA	–	✓	–	Neck attention mechanism replacement only
YOLOv10 + Shape-IoU	–	–	✓	Bounding box loss function replacement only
Full model	✓	✓	✓	Joint optimization of three modules

Values shown in bold are from the Full model with all modules enabled.

The ablation experiments followed these design principles:

1. Variable isolation: only one improvement module was introduced per experiment to prevent multivariate coupling and attribution ambiguity;
2. Data partitioning: complete separation between training and validation sets to eliminate data leakage risks;
3. Hyperparameter freezing: learning rate, batch size, and other parameters strictly aligned with baseline models, with modifications limited to target modules.

#### 3.3.4 Comparative experiment design

To validate the competitiveness of the improved model against existing object detection methods, six mainstream detection models were selected as baseline comparisons.

Table 8 lists the models and configurations used in comparative experiments.

**Table 8:** Ablation experiment module configuration.

Model	Version/configuration	Characteristics
YOLOv10	Official default	Single-stage, NMS-free design
YOLO11	Pre-release version	Introduces region attention mechanism
YOLOv5	YOLOv5n	Industrial lightweight baseline
YOLOv8	YOLOv8n	Multi-task alignment optimization
YOLOv9-t	Tiny configuration	Recursive attention module
SSD	MobileNet-SSD	Classic two-stage lightweight model

To validate the universality of the proposed improvements, the comparative experiments encompass both longitudinal iterations within the YOLO series and cross-paradigm evaluations with two-stage frameworks. The YOLO family—including YOLOv5 with lightweight CSPNet (Do, 2021), YOLOv8 with task-aligned dynamic assigners (Sohan et al., 2024), YOLOv9-t with recursive gated convolution for feature reuse (Wang C. Y. et al., 2024), and YOLO11 with spatial-channel dual-dimensional dynamic modeling (Khanam and Hussain, 2024)—forms a generational sequence of single-stage detectors that directly parallels our multiscale contextual modeling strategy, establishing a methodological dialogue. SSD, as a representative two-stage detector, highlights the limitations of anchor-based mechanisms and NMS post-processing in heterogeneous lesion scenarios, where anchor presets may lead to matching errors (Liu et al., 2016), thus serving as a critical benchmark to assess the medical adaptability of anchor-free single-stage frameworks. All models were evaluated under strict patient-level data isolation, standardized input resolution (bilinear interpolation + zero-padding to preserve aspect ratios), and identical optimization settings, ensuring that outcome differences purely reflect algorithmic variations.

## 4 Results

### 4.1 Ablation experiments revealing module contributions

To comprehensively evaluate the specific contributions of each improved module to model performance, this study systematically analyzes performance differences between the YOLOv10 baseline model and its variants through ablation experiments.

**Figure 11:**
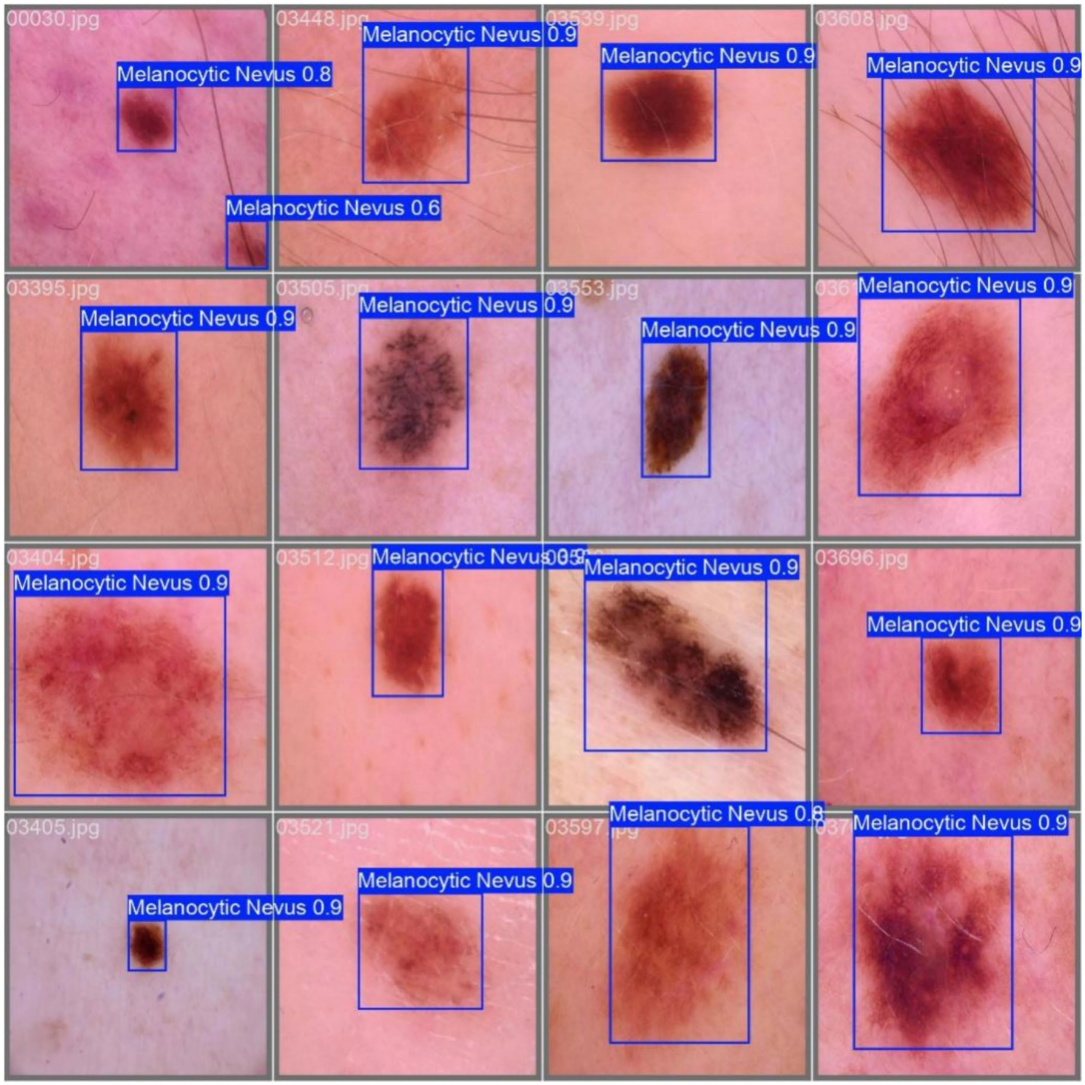
Example detection results.

Example detection results are shown in Figure 11.

Using mAP@0.5 (mean average precision at IoU threshold 0.5) as the core evaluation metric, supplemented by mAP@0.95, Precision (P), Recall (R), model size (Wsz, MB), and inference Latency (ms) we provide a holistic analysis of model performance.

Table 9 presents the performance results of all models.

**Table 9:** Ablation experiment results.

Model	PP-LCNet	MCA	Shape -IoU	Map@0.5 (95% CI)	mAP@0.95 (95% CI)	P	R	Wsz	Latency
YOLOv10 baseline	**–**	**–**	**–**	95.61% (95.30–95.93)	74.77% (74.09–75.47)	92.17%	92.18%	5.51 MB	35.71 ms
YOLOv10 + PP-LCNet	✓	**–**	**–**	97.17% (96.93–97.41)	79.12% (78.55–79.69)	97.28%	93.38%	5.37 MB	32.47 ms
YOLOv10 + MCA	**–**	✓	**–**	96.43% (96.17–96.68)	78.02% (77.45–78.59)	94.21%	91.95%	5.50 MB	33.60 ms
YOLOv10 + shape-IoU	**–**	**–**	✓	96.58% (96.32–96.83)	78.59% (78.02–79.17)	96.04%	89.57%	5.50 MB	33.67 ms
**Full model (all modules)**	✓	✓	✓	**97.69% (97.46–97.91)**	**79.39% (78.83–79.95)**	**94.41%**	**95.65%**	**5.37 MB**	**32.02 ms**

*95% CI calculation: we performed patient-level stratified 10-fold cross-validation. For each metric, the values obtained on the 10 validation folds were combined using a t-distribution (df = 9) to compute the mean ± t₉,0.975 · SD/√10.

Values shown in bold are from the Full model with all modules enabled.

Ablation experiments demonstrate that every module substantially boosts performance. Replacing the backbone with PP-LCNet raises mAP@0.5 from 95.61 to 97.17%, mAP@0.95 to 79.12%, and yields Precision/Recall of 97.28%/93.38%. Introducing the MCA module lifts mAP@0.5 to 96.43%, with Precision 94.21% and Recall 91.95%, proving especially effective for multi-scale targets. Adopting Shape-IoU increases mAP@0.5 and mAP@0.95 to 96.58% and 78.59%, respectively, with Precision 96.04%, evidencing markedly improved boundary fitting. When all three modules are combined, the model achieves an mAP@0.5 of 97.69%—a 2.08-percentage-point gain over the baseline—while mAP@0.95 reaches 79.39% and Precision/Recall balance at 94.41%/95.65%, validating the complementary benefits of PP-LCNet’s feature extraction, MCA’s multi-scale perception, and Shape-IoU’s shape-sensitive regression.

Across all variants the model footprint remains tightly bounded (5.37–5.51 MB), indicating that each module’s parameters are largely offset by PP-LCNet’s channel-sparsity and weight-sharing design. Crucially, inference latency stays below 35 ms on Jetson NX (≈ 29 FPS), with the full model even reaching 32.02 ms—the fastest of all ablation settings despite its highest accuracy. This “performance-for-free” profile means the complete configuration delivers the best trade-off for edge/mobile deployment: it meets real-time thresholds (> 25 FPS), fits comfortably within 8 MB flash budgets, and preserves thermal headroom for continuous operation. If power constraints tighten further, the PP-LCNet-only variant (32.47 ms) offers a graceful fallback with minimal accuracy sacrifice.

### 4.2 Comparative experiments validating model superiority

To validate the superiority of the proposed improved model over existing models, comparative experiments were designed, covering multiple classical object detection models including different versions of the YOLO series (YOLOv10, YOLOv11, YOLOv5, YOLOv8, YOLOv9-t) and the traditional SSD (Single Shot MultiBox Detector). The experimental results are shown in Table 10, where each comparative model is evaluated based on mAP@0.5, mAP@0.95, Precision, and Recall, with mAP@0.5 being the core evaluation metric.

**Table 10:** Ablation experiment results.

Model	mAP@0.5 (95% CI)	mAP@0.95 (95% CI)	*P*	R	Wsz	Latency
YOLOv10	95.61% (95.29–95.93)	74.77% (74.11–75.45)	92.17%	92.18%	5.51 MB	35.71 ms
YOLOv11	94.88% (94.53–95.22)	65.13% (64.21–66.01)	92.62%	89.57%	5.30 MB	35.66 ms
YOLOv5	91.26% (90.88–91.63)	57.69% (56.79–58.60)	88.53%	83.89%	7.25 MB	41.2 ms
YOLOv8	95.39% (95.05–95.72)	62.38% (61.44–63.32)	89.79%	90.00%	5.49 MB	37.8 ms
YOLOv9-t	95.35% (95.01–95.68)	71.27% (70.35–72.16)	97.66%	86.52%	5.98 MB	39.5 ms
SSD	93.33% (93.00–93.64)	69.20% (68.29–70.09)	90.75%	93.64%	14.30 MB	54.7 ms
**Full model (all modules)**	**97.69% (97.46–97.91)**	**79.39% (78.83–79.94)**	**94.41%**	**95.65%**	**5.37 MB**	**32.02 ms**

The meaning of the 95% CI is as follows: under the same data and analysis procedure, if the study were repeated and the interval recalculated each time, about 95% of such intervals would cover the true value of the fold-averaged metric. This interval reflects the uncertainty arising from sample and fold-to-fold variation; it is not equivalent to an individual prediction interval, nor does it guarantee external generalization.

Values shown in bold are from the Full model with all modules enabled.

Comparative results show that baseline YOLOv10 outperforms all other models in melanocytic nevus detection. For example, its mAP@0.5 is 0.73 percentage points higher than that of YOLOv11, with a better precision–recall balance. It exceeds YOLOv5 by 4.35% in mAP@0.5 and 17.08% in mAP@0.95, indicating a clear advantage on small-lesion detection. YOLOv10’s mAP@0.5 is 0.22% higher than YOLOv8’s, reflecting superior fine-grained feature extraction. Although YOLOv10’s precision (92.17%) is slightly below YOLOv9-t’s (97.66%), YOLOv10 achieves higher recall and mAP@0.5. Finally, YOLOv10’s mAP@0.5 is 2.28% higher than SSD’s, underscoring the benefits of an anchor-free framework.

At 5.37 MB in size and 32 ms latency (≈ 31 FPS), the full YOLOv10 variant is both the lightest and the fastest model tested. By contrast, the other models are either heavier (for example, SSD is 14 MB) or slower (YOLOv5 requires 41 ms per image), confirming YOLOv10’s suitability for real-time, on-device screening.

### 4.3 Model robustness analysis

**Figure 12:**
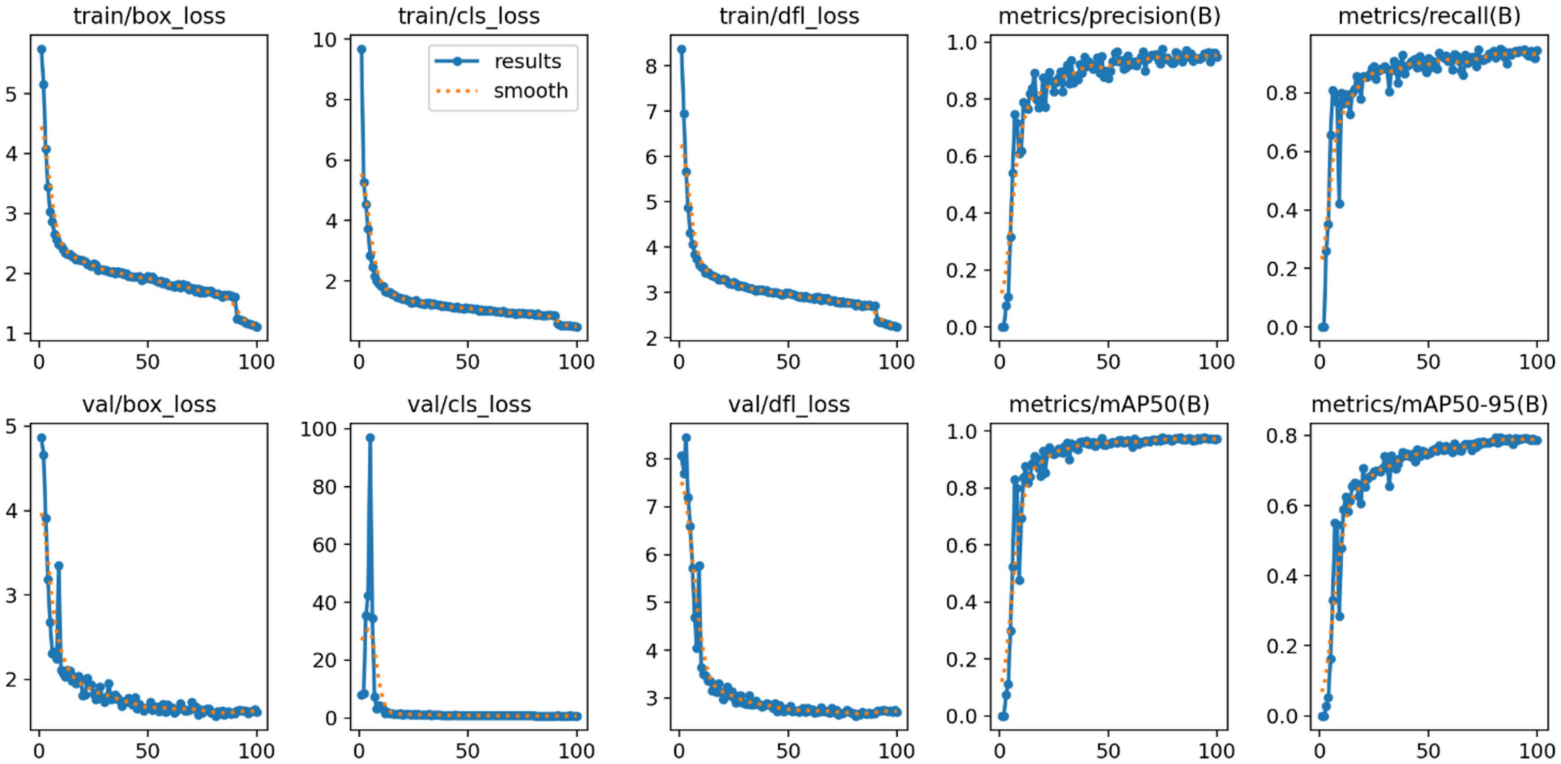
Trends of loss functions and detection performance metrics.

Model robustness is crucial to ensure reliable high-precision performance in melanocytic nevus detection. Performance metrics are illustrated in Figure 12.

We analyzed the model’s loss function curves during training. As shown in Figure 12, the box_loss, cls_loss, and dfl_loss values dropped rapidly in the early epochs and then leveled off at low values. The validation-set loss curves showed a similar decline and closely mirrored the training-set curves, with only minimal differences. This indicates that no significant overfitting or underfitting occurred. Such rapid, stable convergence demonstrates the effectiveness of the model’s optimization process.

Trends in the Precision and Recall metrics further demonstrate the model’s robustness. As shown in Figure 12, Precision rose quickly in the initial training stages and then stabilized above 0.95 with very little fluctuation. Similarly, Recall improved rapidly and plateaued at a level above 0.90. The fact that both metrics remained consistently high indicates that the model effectively detects most melanocytic nevi (high recall) while rarely misclassifying benign lesions as malignant (high precision). These outcomes verify the model’s stable performance and reliability in practical use.

Additionally, the mean Average Precision metrics (mAP@0.5 and mAP@0.5–0.95) showed a similarly stable pattern. As illustrated in Figure 12, mAP@0.5 climbed quickly at the start and then reached a high plateau at roughly 0.98 with minimal fluctuation. The stricter mAP@0.5–0.95 metric also rose steadily and eventually leveled off around 0.80. These observations confirm that the model maintains high localization accuracy even under more stringent IoU thresholds, reinforcing its overall robustness and precise boundary fitting capability.

In summary, the loss curves and evaluation metrics highlight the model’s robust, high-level performance in detecting melanocytic nevi. This stable and reliable performance provides a critical technical foundation for implementing the reverse-exclusion melanoma strategy in clinical practice.

## 5 Clinical reverse exclusion validation

This chapter aims to validate the effectiveness and reliability of the reverse exclusion strategy-based model in melanoma screening.

### 5.1 Research objective and logical premise

This chapter evaluates the clinical safety of the proposed “melanocytic nevus recognition + reverse exclusion” strategy. Since the model exclusively identifies the benign feature “melanocytic nevus,” the screening logic follows:

If the model classifies a case as “melanocytic nevus,” it is deemed “low-risk” and biopsy is not recommended.

Otherwise, the case is categorized as “non-nevus” and classified as high-risk, requiring further intervention.

Under this framework, the definition of false negative (FN) is inverted: “misidentifying melanoma as melanocytic nevus” (erroneously classifying malignant as benign), which carries significant clinical risks.

Therefore, false negative rate (FNR) serves as the core evaluation metric to determine whether this reverse exclusion strategy meets sufficient safety criteria (i.e., FNR ≤ 0.5%).

### 5.2 Model detection process

The experiment utilizes the independent external test set constructed in Chapter 4, comprising 365 dermoscopic images of melanoma confirmed by biopsy, with no melanocytic nevi or other benign lesions, ensuring unambiguous classification.

All images undergo standardized normalization and are processed through the improved model for inference. The model exclusively detects the “melanocytic nevus” category:

Detection bounding boxes indicate “melanocytic nevus” classification.
Absence of bounding boxes indicates “non-melanocytic nevus” classification.

### 5.3 Research objective and logical premise

According to the clinical inverse exclusion definition, the false negative rate (FNR) is calculated as Equation 8 (Bossuyt et al., 2003):

(8) $$\mathrm{FNR}=\frac{\mathrm{FN}}{\mathrm{TP}+\mathrm{FN}}$$

where, FN (false negative): misclassifying melanoma as melanocytic nevus (i.e., labeling malignancy as benign); TP (true positive): correctly classifying melanoma as “non-melanocytic nevus” (i.e., identifying high-risk lesions).

### 5.4 Experimental results

**Figure 13:**
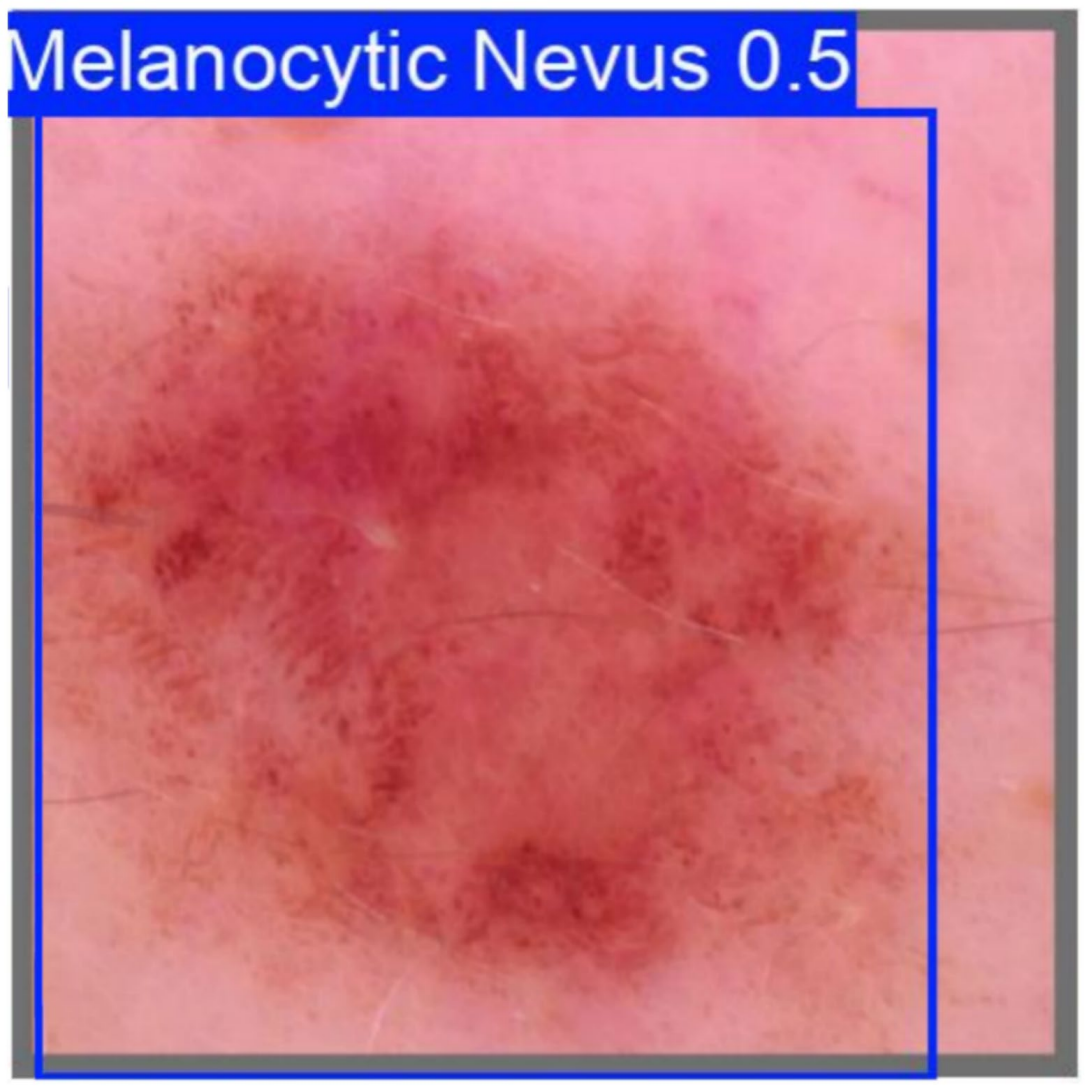
Misclassified sample.

Among the 365 melanoma samples, the model failed to detect melanocytic nevus in 364 cases (TN), while misclassifying 1 case as nevus (FN) with a detection confidence of 0.5. The misclassified sample is shown in Figure 13

Table 11 shows the test statistics.

**Table 11:** Inverse detection statistics on melanoma dataset.

Category	Count	Description
Total melanoma images	365	All biopsy-confirmed melanomas
Classified as “Non-Nevus” (TN)	364	Correctly excluded
Classified as “Nevus” (FN)	1	Misclassified as nevus (confidence 0.5)
False Negative Rate (FNR)	0.27%	Below clinical s

### 5.5 Morphological analysis of the misclassified lesion

The dermoscopic image mislabelled by the network as a benign melanocytic naevus exhibits several geometric and topological hallmarks of early melanoma that escape the current model.

**Figure 14:**
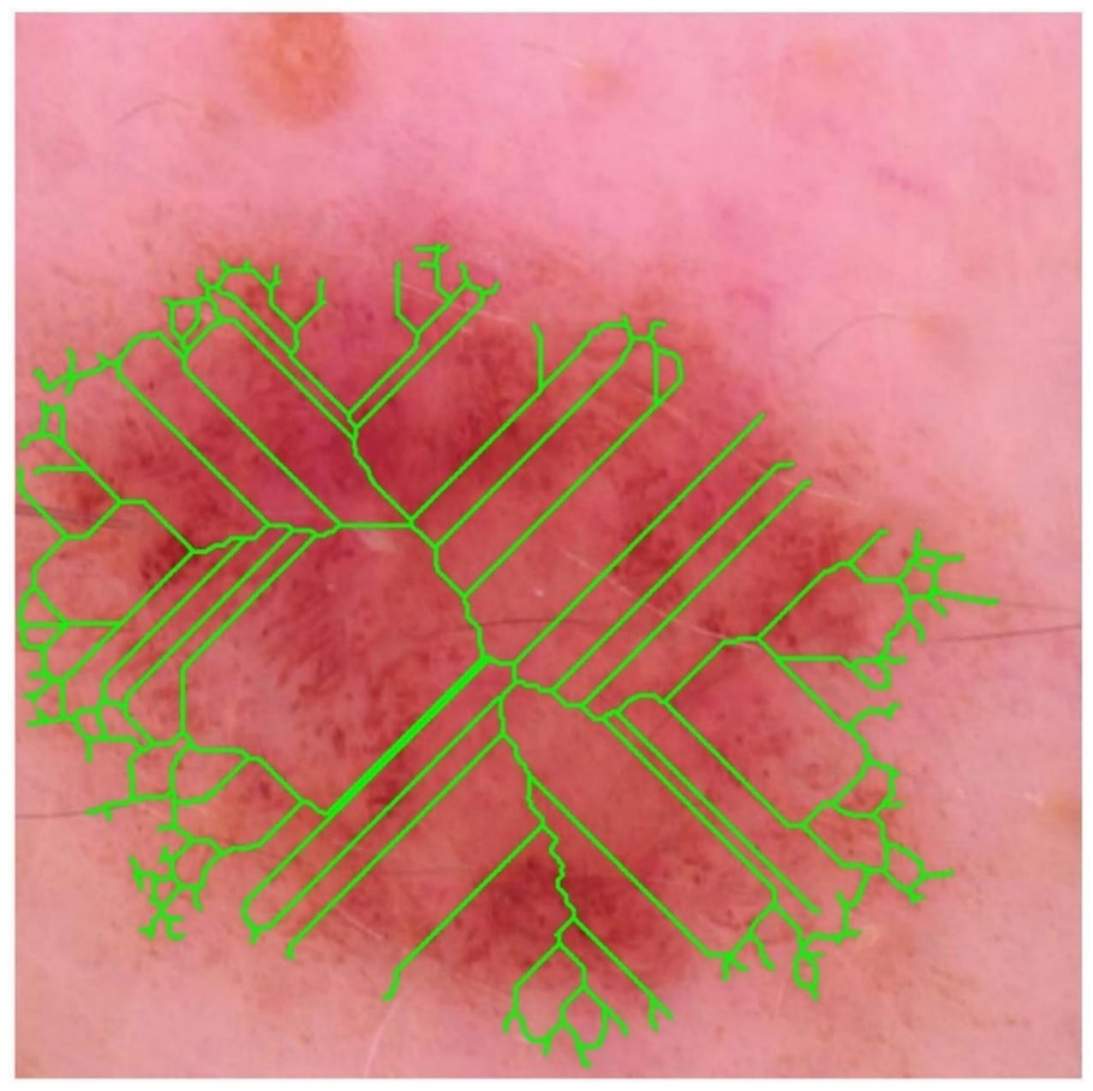
Skeleton overlay.

The binary mask was thinned using the Zhang–Suen algorithm, and the resulting skeleton (lime) is super-imposed on the dermoscopic image (see Figure 14). High branch density and numerous terminal nodes indicate irregular peripheral outgrowth and suggest satellite invasion beyond the main tumour mass.

**Figure 15:**
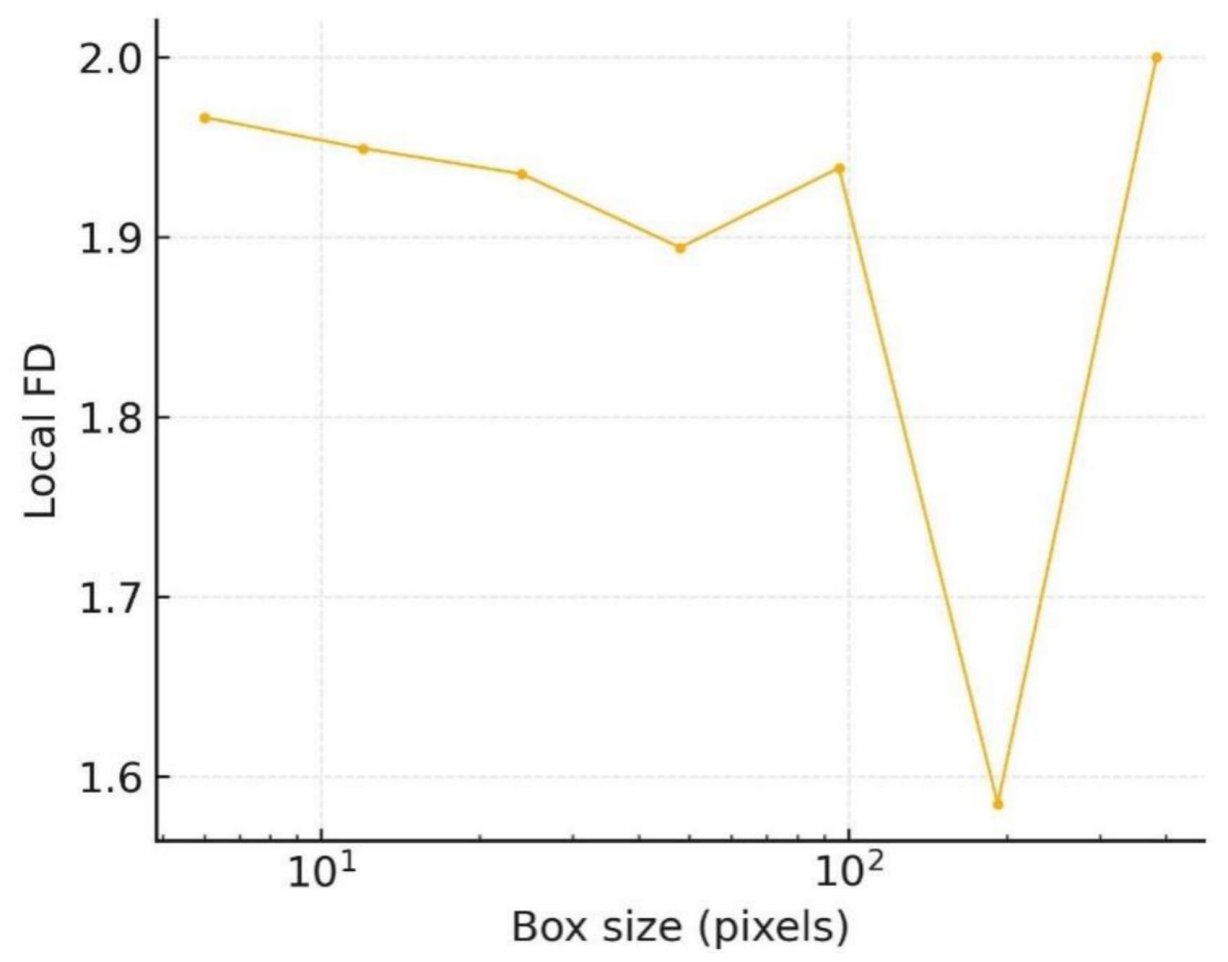
Local FD spectrum.

The scale-resolved spectrum in Figure 15 refines this observation: between 16 px and 64 px the local fractal dimension stays in the 1.92–1.95 range, while beyond 128 px it abruptly approaches 2.0. These twin plateaux indicate the coexistence of coarse lobular bulges and fine spiculate structures, an architectural pattern frequently reported in early invasive melanoma.

**Figure 16:**
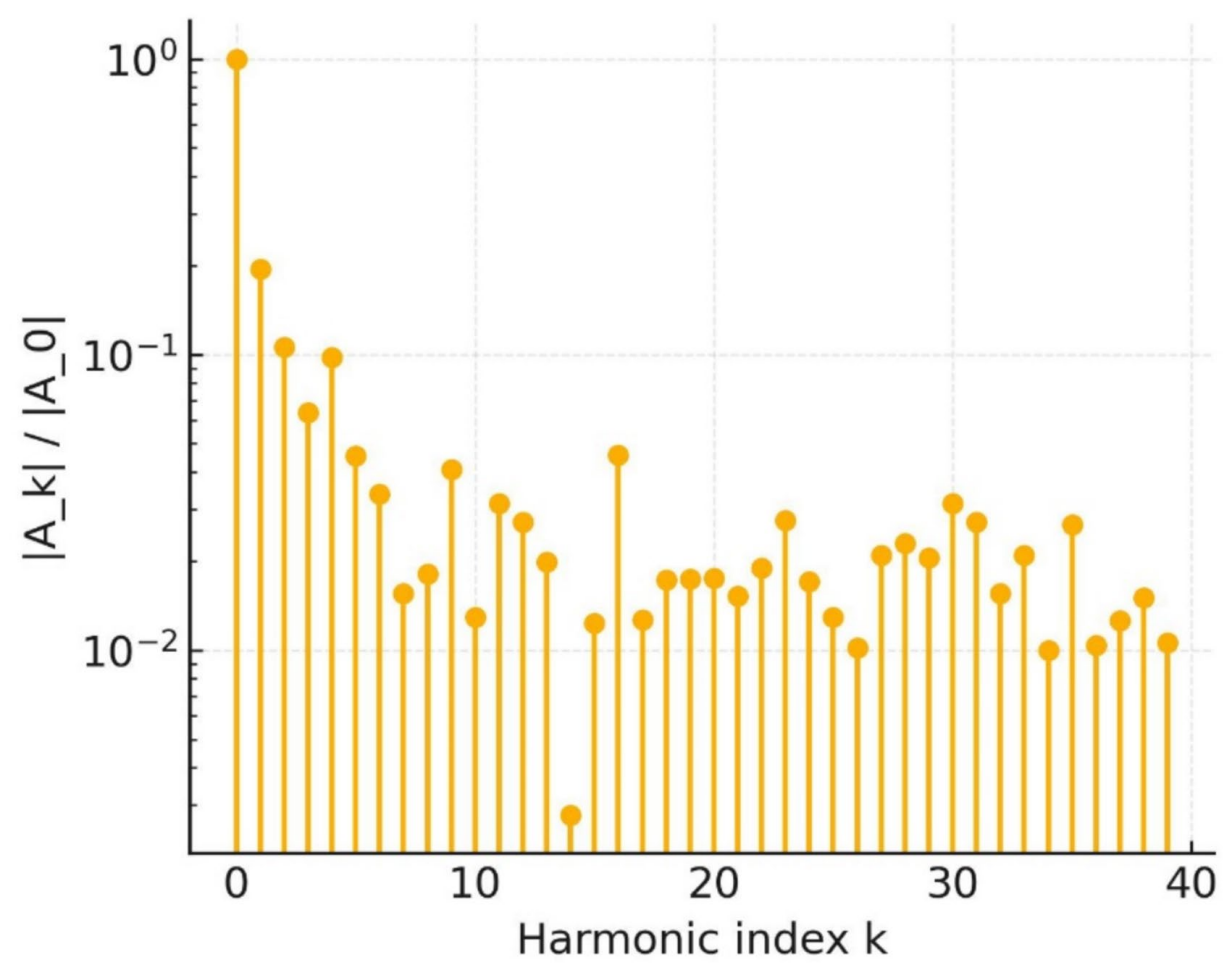
EFD magnitude.

Shape frequency analysis supports this conclusion. The elliptic Fourier magnitude spectrum in Figure 16 decays only slowly up to the 20th harmonic; every component above the third harmonic exceeds 10^−2^, whereas benign contours typically fall one to two orders of magnitude below that threshold. The preserved high-frequency power confirms the abundance of micro-indentations already visible in the raw outline.

**Figure 17:**
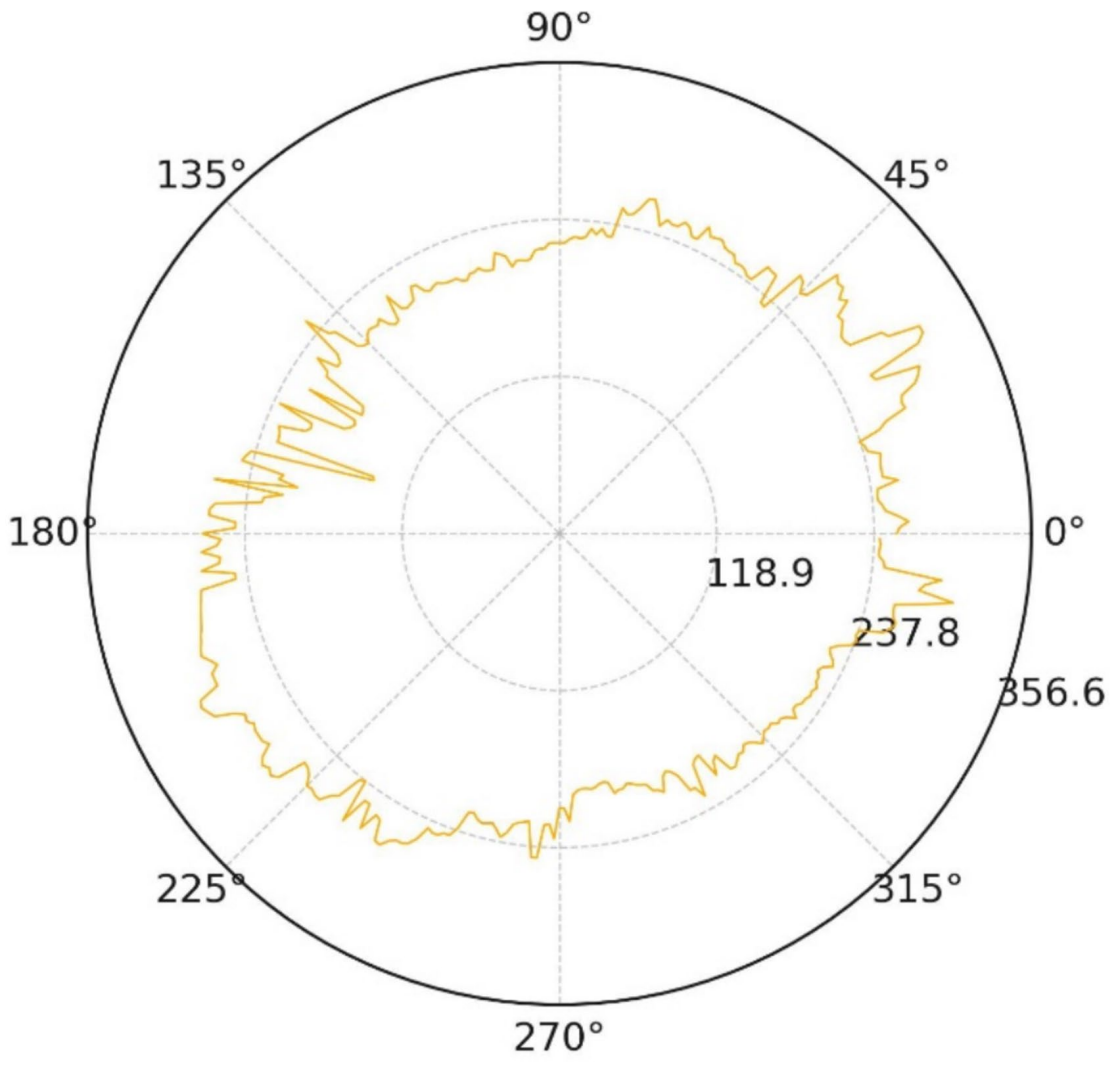
Radial distance profile.

Geometric symmetry is equally compromised. The polar radial-distance trace (Figure 17) oscillates by roughly 32% of the mean radius, with pronounced peaks along the 60°–120° and 240°–300° directions.

**Figure 18:**
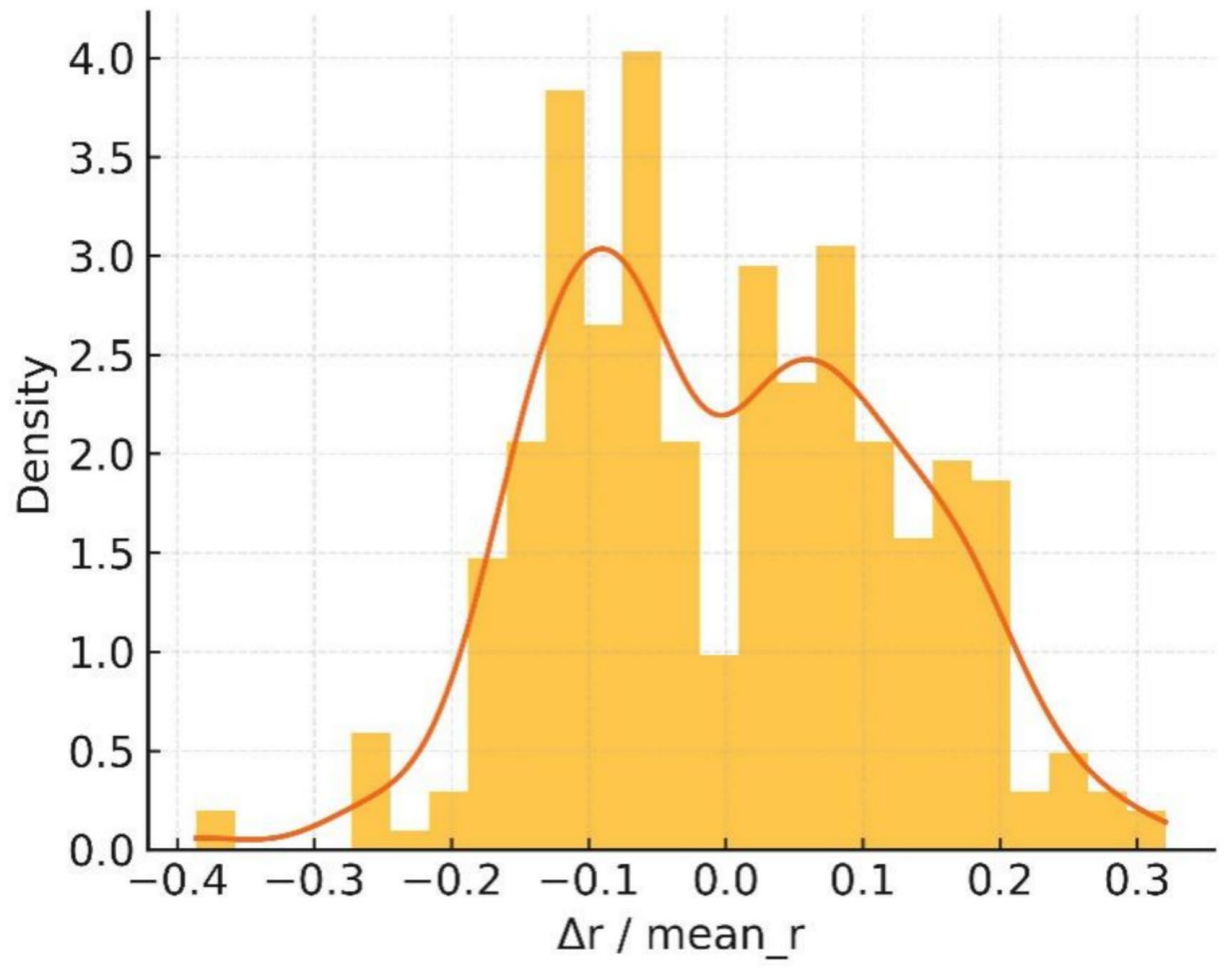
Δr/r̄ histogram.

The normalised deviation histogram in Figure 18 is clearly bimodal: about 41% of boundary pixels deviate from the mean radius by more than 10%, whereas benign controls rarely exceed 15%. These measurements satisfy the asymmetry clause of the ABCDE rule and reinforce the malignant impression.

Taken together, the five quantitative views demonstrate excessive boundary complexity, multiscale serration and pronounced bilateral asymmetry—three independent morphological signatures of melanoma. The convolutional network nevertheless produced a benign probability of 0.5, implying that its decision was dominated by homogeneous central pigmentation while largely ignoring higher-order geometric cues.

### 5.6 Benchmarking false-negative rate (FNR) against dermatologists using the ABCDE rule and 7-point checklist

In early melanoma screening, the clinical *gold standard* remains the visual assessment of experienced dermatologists, supported by two guideline-endorsed scoring systems—the ABCDE rule and the 7-point checklist. Demonstrating a clear performance advantage over these real-world decision benchmarks is essential for establishing the safety and utility of any AI model. Accordingly, this section benchmarks the enhanced YOLOv10 framework against dermatologists operating within these two scoring paradigms, focusing on the critical safety metric of false-negative rate (FNR).

To eliminate memory and ordering bias, three dermatologists with ≥10 years of experience independently interpreted the same set of 365 histopathology-confirmed melanoma images in a double-blind, two-round protocol. In the first round, only the ABCDE rule was applied; after a four-week washout period, the image order was re-randomized and the second round was conducted using only the 7-point checklist. Pathology results and model outputs were concealed from all readers. The comparative results are summarized in Table 12.

**Table 12:** False-negative counts and FNRs for model and dermatologist scoring systems.

Method	FN	FNR
Enhanced YOLOv10	1	0.27%
Dermatologists – ABCDE	41	11.23%
Dermatologists – 7-point	46	12.60%

The model missed only one melanoma among 365 cases, yielding an FNR roughly forty-fold lower than that of dermatologists using either conventional scoring system (McNemar *p* < 0.001). This marked reduction highlights the model’s superior diagnostic safety margin, particularly in minimizing false-negative outcomes in early melanoma screening.

## 6 Conclusion

This study addresses the persistent issue of missed melanoma diagnoses by proposing a novel “benign-first, reverse-exclusion” workflow. In this approach, the model first confidently identifies melanocytic nevi and then infers that melanoma is absent. Within the YOLOv10 framework, we introduced three specific upgrades: (i) a lightweight PP-LCNet backbone to combat the loss of fine (sub-3 mm) texture detail, (ii) a Multiscale Contextual Attention (MCA) module to improve cross-scale feature fusion, and (iii) a shape-aware Shape-IoU loss for more accurate boundary fitting.

On a multi-centre benign nevus dataset, the enhanced detector achieved mAP@0.5 of 97.69% and a precision of 0.94 with recall of 0.96. Most critically, on an independent cohort of 365 biopsy-proven melanomas, the false-negative rate (FNR) was only 0.27%, well below the clinically accepted 0.5% ceiling. In practical terms, out of 1,000 screened lesions the system would be expected to miss at most 3 melanomas — far fewer than conventional visual exams or generic AI tools — thereby preserving the life-saving window afforded by early detection.

Beyond accuracy, the model also meets two important clinical requirements: (1) an inference latency below 35 ms on edge devices, which enables real-time dermoscopy in clinics or mobile screening vans; and (2) a compact 5.4 MB model size, small enough for deployment on a smartphone — lowering the barrier to broad adoption in primary care settings.

The loss curves converged rapidly and smoothly, and the precision–recall and F1–confidence curves flattened into stable plateaus across a wide range of confidence thresholds. Together, these outcomes demonstrate that the detector maintains a low FNR and high accuracy under a variety of deployment conditions.

Three limitations remain. First, our training and test cohorts consisted exclusively of East-Asian patients with Fitzpatrick type II–III skin, so a multi-ethnic validation is essential before any global roll-out. Second, because our image archive was frozen as of 31 December 2024, only a small fraction of cases have reached the 5-year follow-up mark commonly used to assess melanoma recurrence. A prospective registry linking baseline dermoscopic images to outcomes ≥5 years is being established to quantify real-world reductions in unnecessary biopsies and to ensure long-term safety. Third, before large-scale clinical deployment, the system must meet emerging regulatory and ethical standards. Obtaining CE marking in Europe or FDA clearance in the US will depend on demonstrating robust performance, instituting post-market surveillance plans, and — critically — ensuring the model’s explainability. Although the reverse-exclusion logic is clinically intuitive, future work should integrate formal interpretability tools (for example, class-activation heatmaps or token-level attention visualizations) and produce regulatory-grade documentation to address algorithmic bias, cybersecurity, and data privacy compliance.

In conclusion, by anchoring an ultra-low FNR of 0.27% as its safety floor and tightly coupling deep-learning advances with the reverse-exclusion paradigm, the proposed detector offers a practical, efficient, and safe solution for early melanoma diagnosis and provides a foundation for wider AI-assisted skin-cancer screening.

## Data Availability

The raw data supporting the conclusions of this article will be made available by the authors, without undue reservation.

## References

[ref1] Akash N. R. J. Kaushik A. Sivaswamy J., (2023). Evidence-driven differential diagnosis of malignant melanoma. In Proceedings of the ISIC workshop, 1–8.

[ref2] Almubarak H. A. Stanley R. J. Stoecker W. V. Moss R. H. (2017). Fuzzy color clustering for melanoma diagnosis in dermoscopy images. Information 8:89. doi: 10.3390/info8030089

[ref3] Alzamili A. H. Ruhaiyem N. I. R. (2025). A comprehensive review of deep learning and machine learning techniques for early-stage skin cancer detection: challenges and research gaps. J. Intell. Syst. 34:20240381. doi: 10.1515/jisys-2024-0381

[ref4] Annadatha S. Hua Q. Fridberg M. Lindstrøm Jensen T. Liu J. Kold S.. (2022). Preparing infection detection technology for hospital-at-home after lower-limb external fixation. Digit. Health. 8:20552076221109502. doi: 10.1177/20552076221109502, PMID: 35783467 PMC9243585

[ref37] Arumi-Uria M. McNutt N. S. Finnerty B. (2003). Grading of atypia in nevi: correlation with melanoma risk. Mod. Pathol. 16, 764–771. doi: 10.1097/01.MP.0000082394.91761.E5 12920220

[ref5] Bossuyt P. M. Reitsma J. B. Bruns D. E. Gatsonis C. A. Glasziou P. P. Irwig L. M.. (2003). The STARD statement for reporting studies of diagnostic accuracy: explanation and elaboration. Ann. Intern. Med. 138, W1–W12. doi: 10.7326/0003-4819-138-1-200301070-00012-W1, PMID: 12513067

[ref6] Cai D. Zhang Z. Zhang Z. (2023). Corner-point and foreground-area IoU loss: better localization of small objects in bounding-box regression. Sensors 23:4961. doi: 10.3390/s23104961, PMID: 37430876 PMC10223589

[ref19] Carrera C. Segura S. Aguilera P. Scalvenzi M. Longo C. Barreiro A.. (2017a). Dermoscopic clues for diagnosing melanomas that resemble seborrheic keratosis. JAMA Dermatol. 153, 544–551. doi: 10.1001/jamadermatol.2017.0129 28355453 PMC5540029

[ref7] Carrera C. Segura S. Aguilera P. Takigami C. M. Gomes A. Barreiro A.. (2017b). Dermoscopy improves the diagnostic accuracy of melanomas clinically resembling seborrheic keratosis: a cross-sectional study. Dermatology 233, 471–479. doi: 10.1159/000486851 29502116

[ref8] Codella N. C. F. Gutman D. Celebi M. E. Helba B. Marchetti M. A. Dusza S. W.. (2017). Skin lesion analysis toward melanoma detection: a challenge at ISBI 2017 (ISIC). arXiv [Preprint]. doi: 10.48550/arXiv.1710.05006

[ref9] Cui C. Gao T. Wei S. Du Y. Guo R. Dong S.. (2021). PP-lcnet: a lightweight CPU convolutional neural network. arXiv [Preprint]. doi: 10.48550/arXiv.2109.15099

[ref10] Dinnes J. Deeks J. J. Chuchu N. Ferrante di Ruffano L. Matin R. N. Thomson D. R.. (2018). Dermoscopy, with and without visual inspection, for diagnosing melanoma in adults. Cochrane Database Syst. Rev. 12:CD011902. doi: 10.1002/14651858.CD011902.pub2, PMID: 30521682 PMC6517096

[ref11] Do T. (2021). Evolution of YOLO algorithm and YOLOv5: The state-of-the-art object detection algorithm. Oulu: Oulu University of Applied Sciences.

[ref12] Esteva A. Kuprel B. Novoa R. Novoa R. A. Ko J. Swetter S. M.. (2017). Dermatologist-level classification of skin cancer with deep neural networks. Nature 542, 115–118. doi: 10.1038/nature21056, PMID: 28117445 PMC8382232

[ref13] Ferris L. K. Harris R. J. (2012). New diagnostic aides for melanoma. Dermatol. Clin. 30, 535–545. doi: 10.1016/j.det.2012.04.012, PMID: 22800557 PMC3623959

[ref14] Fikrle T. Pizinger K. (2007). Border sharpness of benign nevi in dermoscopic images. J. Eur. Acad. Dermatol. Venereol. 21, 48–55. doi: 10.1111/j.1468-3083.2006.01864.x 17207167

[ref15] Flosdorf C. Engelker J. Keller I. Mohr N. (2024). Skin Cancer detection utilizing deep learning: classification of skin lesion images using a vision transformer. arXiv. doi: 10.48550/arXiv.2407.18554

[ref16] Flotte T. J. Lamberti L. L. (1992). Symmetry features of melanocytic nevi. Mod. Pathol. 5, 333–336. doi: 10.1007/BF03000228 1495938

[ref17] Henning J. S. Dusza S. W. Wang S. Q. Marghoob A. A. Rabinovitz H. S. Polsky D.. (2007). The CASH algorithm for dermoscopy. J. Am. Acad. Dermatol. 56, 45–52. doi: 10.1016/j.jaad.2006.09.003 17190620

[ref18] Hunziker M. F. V. Abdalla B. M. Z. Brandão F. V. Meneghello L. P. Hunnicutt J. M. S. di Giacomo T. H. B.. (2023). Exploring small-diameter melanomas: a retrospective study on clinical and Dermoscopic features. Life 13:1907. doi: 10.3390/life13091907, PMID: 37763310 PMC10533118

[ref20] Khanam R. Hussain M. (2024). YOLOv11: an overview of the key architectural enhancements. arXiv:2410.17725. doi: 10.48550/arXiv.2410.17725

[ref21] Krizhevsky A. Sutskever I. Hinton G. E. (2017). Imagenet classification with deep convolutional neural networks. Commun. ACM 60, 84–90. doi: 10.1145/3065386

[ref22] Lallas A. Apalla Z. Chaidemenos G. (2012). New trends in dermoscopy to minimise the risk of missing melanoma. J. Skin Cancer 2012:820474. doi: 10.1155/2012/820474, PMID: 23094161 PMC3472511

[ref23] Liu W. Anguelov D. Erhan D. Szegedy C. Reed S. Fu C.-Y.. (2016). “SSD: Single shot MultiBox detector,” in Computer Vision – ECCV. eds. Leibe B. Matas J. Sebe N. Welling M. (Cham: Springer), 21–37.

[ref24] Liutkus J. Kriukas A. Stragyte D. Mazeika E. Raudonis V. Galetzka W.. (2023). Accuracy of a smartphone-based YOLO application for classification of melanomas, melanocytic nevi, and seborrheic keratoses. Diagnostics 13:2139. doi: 10.3390/diagnostics13132139 37443533 PMC10340832

[ref25] Naseri H. Safaei A. A. (2025). Diagnosis and prognosis of melanoma from Dermoscopy images using machine learning and deep learning: a systematic literature review. BMC Cancer 25:75. doi: 10.1186/s12885-024-13423-y, PMID: 39806282 PMC11727731

[ref27] Nguyen E. H. Yang H. Deng R. Lu Y. Zhu Z. Roland J. T.. (2022). Circle representation for medical object detection. IEEE Trans. Med. Imaging 41, 746–754. doi: 10.1109/TMI.2021.3122835, PMID: 34699352 PMC8963364

[ref29] Rotemberg V. Kurtansky N. Betz-Stablein B. Caffery L. Chousakos E. Codella N.. (2021). A patient-centric dataset of images and metadata for identifying melanomas using clinical context. Sci Data 8:34. doi: 10.1038/s41597-021-00815-z, PMID: 33510154 PMC7843971

[ref28] Seidenari S. Pellacani G. Grana C. (2005). Pigment distribution in melanocytic lesion images: a digital parameter to be employed for computer‐aided diagnosis. Skin Res Technol. 11, 236–241. doi: 10.1111/j.0909-725X.2005.00123.x 16221139

[ref30] Shen S. Xu M. Zhang F. Shao P. Liu H. Xu L.. (2021). Low-cost and high-performance data augmentation for deep-learning-based skin lesion classification. arXiv [Preprint]. doi: 10.48550/arXiv.2101.02353 PMC10521644 37850173

[ref31] Sohan M. Ram S. T. Reddy V. R. C. V. (2024). “A review on YOLOv8 and its advancements,” in Data intelligence and cognitive informatics. eds. Jacob I. J. Piramuthu S. Falkowski-Gilski P. (Singapore: Springer), 529–545.

[ref32] Song W. Wang S. Aortic aneurysm detection in CT medical images based on YOLOv5. Acad. J. Comput. Inf. Sci. (2025), 8, 56–63. Available online at: https://francis-press.com/papers/18603

[ref26] Stell V. H. Norton H. J. Smith K. S. Salo J. C. White Jr R. L. (2007). Method of biopsy and incidence of positive margins in primary melanoma. Ann. Surg. Oncol. 14, 893–898. doi: 10.1245/s10434-006-9240-4 17119869

[ref33] Stevenson A. D. Mickan S. Mallett S. Ayya M. (2013). Systematic review of diagnostic accuracy of reflectance confocal microscopy for melanoma diagnosis in clinically equivocal lesions. Dermatol. Pract. Concept. 3, 19–27. doi: 10.5826/dpc.0304a05 24282659 PMC3839827

[ref34] Thomas L. Tranchand P. Berard F. Secchi T. Colin C. Moulin G. (1998). Semiological value of ABCDE criteria in the diagnosis of cutaneous pigmented tumors. Dermatology 197, 11–17. doi: 10.1159/000017969, PMID: 9693179

[ref35] Tsao H. Olazagasti J. M. Cordoro K. M. Brewer J. D. Taylor S. C. Bordeaux J. S.. (2015). Early detection of melanoma: reviewing the ABCDEs. J. Am. Acad. Dermatol. 72, 717–723. doi: 10.1016/j.jaad.2014.01.887 25698455

[ref36] Tschandl P. Rinner C. Apalla Z. Argenziano G. Codella N. Halpern A.. (2020). Human–computer collaboration for skin cancer recognition. Nat. Med. 26, 1229–1234. doi: 10.1038/s41591-020-0942-0 32572267

[ref38] Van Loo E. Sinx K. A. E. Welzel J. Schuh S. Kelleners-Smeets N. W. J. Mosterd K.. (2020). Cumulative-sum analysis for the learning curve of optical coherence tomography-assisted diagnosis of basal cell carcinoma. Acta Derm. Venereol. 100:adv00343. doi: 10.2340/00015555-3696, PMID: 33205824 PMC9309705

[ref39] Wang A. Chen H. Liu L. Chen K. Lin Z. Han J. (2024). YOLOv10: real-time end-to-end object detection. arXiv [Preprint]. doi: 10.48550/arXiv.2405.14458

[ref40] Wang C. Y. Yeh I. H. Liao H. Y. M. (2024). “YOLOv9: learning what you want to learn using programmable gradient information,” in Computer vision – ECCV 2024; *LNCS 15089*. eds. Leonardis A. Ricci E. Roth S. Russakovsky O. Sattler T. Varol G. (Cham: Springer), 1–21.

[ref41] Whited J. D. Hall R. P. Simel D. L. Horner R. D. (1997). Primary-care clinicians’ performance for detecting actinic keratoses and skin cancer. Arch. Intern. Med. 157, 985–990. doi: 10.1001/archinte.157.9.985, PMID: 9140269

[ref42] Ye R. Shao G. He Y. Gao Q. Li T. (2024). YOLOv8-RMDA: lightweight YOLOv8 network for early detection of small target diseases in tea. Sensors 24:2896. doi: 10.3390/s24092896, PMID: 38733002 PMC11086262

[ref43] Yu Y. Zhang Y. Cheng Z. Song Z. Tang C. (2023). MCA: multidimensional collaborative attention in deep convolutional neural networks for image recognition. Eng. Appl. Artif. Intell. 126:107079. doi: 10.1016/j.engappai.2023.107079

[ref44] Zhang H. Zhang S. (2023). Shape-iou: more accurate metric considering bounding-box shape and scale. arXiv. doi: 10.48550/arXiv.2312.17663

